# Modulation of corneal sensory processing and pain responses by dry eye and corneal wounding

**DOI:** 10.3389/fncel.2026.1767673

**Published:** 2026-06-02

**Authors:** Ebru Yaman, Yangluowa Qu, Kaylin C. Arpaia, Fairouz Elsaeidi, Uma Lakshmi Balakrishnan, Lance He, Henry Skrehot, Jehan Alam, Rui Chen, Mary Ann Stepp, Cintia S. de Paiva, Stephen C. Pflugfelder

**Affiliations:** 1 Ocular Surface Center, Department of Ophthalmology, Cullen Eye Institute, Baylor College of Medicine, Houston, TX, United States; 2 Baylor College of Medicine, School of Medicine, Houston, TX, United States; 3 Department of Ophthalmology and Visual Sciences, Gavin Herbert Eye Institute, University of California Irvine School of Medicine, Irvine, CA, United States; 4 The George Washington University School of Medicine and Health Sciences, Washington, DC, United States

**Keywords:** corneal epithelial barrier, corneal sensory dysfunction, dry eye disease, MMP-9, ocular pain, TRPM8, TRPV1

## Abstract

**Background:**

Dry eye disease (DED) is a multifactorial disorder of the ocular surface in which tear film instability, epithelial barrier disruption, and neurosensory dysfunction contribute to symptom generation. Although several contributing factors have been identified, how different forms of ocular surface stress affect sensory pathways and behavioral responses remains unclear.

**Purpose:**

This study aimed to evaluate corneal barrier function, sensory responses, and pain-related behaviors across acute and chronic dry eye and corneal injury models, and to assess stimulus-specific responses associated with TRPV1 and TRPM8 mediated pathways.

**Methods:**

Female C57BL/6 J wild type and Mmp9KO mice were evaluated in acute desiccating stress (DS), chronic [lacrimal gland excision (LGE) and aging], and corneal epithelial debridement models. Tear production, corneal barrier integrity, and mechanical and chemical sensitivity were assessed using established assays. Behavioral responses, including palpebral aperture height–width ratio (HWR), blink frequency, and pawing, were quantified following stimulation with capsaicin, hypertonic saline, and menthol. Human dry eye subjects were analyzed using video based measurements of HWR and blink frequency.

**Results:**

Acute and chronic dry eye models and patients showed reduced palpebral aperture and decreased mechanical sensitivity. Sensitivity to CO₂ gas was unchanged, except in Mmp9KO. Capsaicin, hypertonic saline, and menthol reduced HWR in acute dry eye, with the greatest effect from capsaicin. Chronic dry eye showed reduced sensitivity to capsaicin and no change to hypertonic saline. Corneal debridement reduced mechanical sensitivity but increased responses to chemical stimulation.

**Conclusion:**

Reduced mechanical corneal sensitivity and palpebral aperture HWR represent consistent sensory phenotypes across dry eye models and patients, supporting translational relevance. Divergent responses to chemical stimuli between acute and chronic dry eye suggest neurosensory processing evolves with disease chronicity and engages distinct sensory pathways.

## 1 Introduction

Dry eye disease (DED) is a common multifactorial disorder of the ocular surface that affects hundreds of millions of people worldwide and significantly impairs quality of life (Sharma and Hindman, 2014; Stapleton et al., 2017). In the United States alone, approximately 5–15% of the population (16.7–50.2 million individuals) are affected, with prevalence rising sharply in individuals over 50 years of age and in women (McCann et al., 2022; Schaumberg et al., 2003; Stapleton et al., 2017). DED is characterized by tear film instability, epithelial barrier disruption, and ocular discomfort that frequently progresses to neuropathic pain (Craig et al., 2017; Stapleton et al., 2017). Although inflammation and tear deficiency are central features of DED, increasing evidence highlights the importance of altered sensory function in disease pathogenesis (Belmonte et al., 2015; Galor et al., 2018).

The cornea is among the most densely innervated tissues in the body, and corneal nerves critically regulate blink reflexes, tear secretion, and epithelial homeostasis (Müller et al., 2003). Disruption of these pathways contributes to both the symptoms and progression of DED. However, how different forms of environmental stress, aging, epithelial injury, and genetic susceptibility alter corneal sensory function and barrier integrity remains incompletely understood.

Experimental mouse models provide key mechanistic insights into ocular surface biology and allow controlled dissection of these interacting factors. Desiccating stress. (DS) has been widely used to mimic environmental drivers of human dry eye, particularly the combination of tear film hyperosmolarity and low humidity that exacerbate ocular surface damage (Dursun et al., 2002; Stern and Pflugfelder, 2017). This model reflects clinical situations in which patients are exposed to air drafts or reduced ambient humidity, such as office or desert environments, and is considered one of the most relevant translational paradigms for evaporative dry eye (Huang et al., 2021).

Lacrimal gland excision (LGE) is a surgical approach that eliminates aqueous tear production and provides a robust model of aqueous-deficient dry eye (Mecum et al., 2020). Clinically, this parallels severe aqueous deficiency states such as Sjögren’s syndrome or glandular damage following radiation therapy. By including both LGE and sham-operated groups, it is possible to directly assess the contribution of lacrimal gland function to tear homeostasis and sensory responses.

Aging is another critical factor, as elderly patients frequently experience reduced tear production, epithelial barrier compromise, diminished corneal sensitivity, and immune-mediated ocular surface changes (McClellan et al., 2014; Sharma and Hindman, 2014). Studying aged mice allows direct modeling of physiological decline that parallels age-related dry eye seen in the clinic (Abu-Romman et al., 2024).

Mice lacking *Mmp9* provide a genetic model to interrogate the role of inflammatory and extracellular matrix remodeling pathways in ocular surface disease (Pflugfelder et al., 2005). Given that elevated MMP-9 activity is consistently reported in DED and used clinically as a biomarker of disease severity (Lee et al., 2021; Messmer, 2015; Pflugfelder et al., 2005), studying Mmp9KO mice allows us to clarify how the absence of this enzyme alters epithelial integrity and sensory signaling compared to wild-type animals (Chotikavanich et al., 2009).

Corneal epithelial debridement is a reproducible method to study acute epithelial injury and wound healing. Clinically, this mimics corneal abrasions or surgical epithelial removal (e.g., in photorefractive keratectomy), conditions where transient barrier loss can precipitate both pain and altered neural responses (Wilson, 2020).

Transient receptor potential (TRP) channels expressed by corneal afferents mediate chemosensory and thermosensory responses. TRPV1 is activated by noxious chemical and thermal stimuli, including capsaicin, and plays a critical role in ocular surface pain (Hatta et al., 2019; Pizzano et al., 2024). Hyperosmolarity, a hallmark of the DED tear film, predominantly engages TRPV1-associated polymodal nociceptors, while also involving additional sensory pathways, thereby linking desiccating stress to nociceptive signaling (Vereertbrugghen and Galletti, 2022). In contrast, TRPM8 is activated by cooling agents such as menthol and drives basal tear secretion through cold-sensitive afferents (Parra et al., 2010). Dysregulation of these channels has been implicated in both tear film instability and aberrant ocular discomfort in DED (Quallo et al., 2015). Although individual roles of TRPV1 and TRPM8 in corneal sensory signaling have been studied, their functional responses have not been systematically compared across multiple dry eye and corneal injury models.

Topical capsaicin application mimics the neuropathic/inflammatory subtype of dry eye by directly activating TRPV1. Capsaicin, the pungent alkaloid of chili peppers, exerts its effects primarily via binding to the TRPV1, a heat- and proton-gated channel expressed on polymodal nociceptors. Structural studies reveal that capsaicin docks in a “tail-up, head-down” configuration within a hydrophobic pocket formed by the S3–S4 and S4–S5 transmembrane linkers, engaging both hydrogen bonds and van der Waals contacts to stabilize the open state (Domene et al., 2022; Yang et al., 2015). Upon binding, TRPV1 permits Na^+^ and Ca^2+^ influx, depolarizing the nerve terminal and triggering action potentials that convey burning pain. This influx also drives Ca^2+^-dependent release of substance P and CGRP, promoting neurogenic inflammation, increased blink rate, tear-film instability, and hyperosmolar stress, features characteristic of inflammatory dry eye (Hwang et al., 2021; Li et al., 2019). Prolonged or repeated exposure leads to Ca^2+^-dependent desensitization via phosphorylation–dephosphorylation cycles and PIP₂ depletion, underlying the paradoxical analgesic effects of topical capsaicin preparations.

Hyperosmolar solutions are noxious to the corneal surface, activating polymodal nociceptors via cell-shrinkage induced membrane tension and osmolyte-sensitive ion channels. Hyperosmolar solution stimulation provokes rapid release of neuropeptides such as CGRP without causing overt axonal degeneration, indicating engagement of a distinct nociceptor subset or intracellular signaling cascade (Hwang et al., 2021; Li et al., 2019; Ma et al., 2024). Electrophysiological recordings demonstrate altered firing patterns in both cold thermoreceptors and polymodal fibers, suggesting hyperosmolar solution triggers cross-modal sensitization and modulates corneal nerve excitability across multiple TRP pathways (Belmonte et al., 2017; Hirata and Meng, 2010; Kovács et al., 2016).

Menthol, a cooling terpenoid from mint, selectively activates TRPM8, the primary cold-sensing TRP channel. Thermodynamic analyses and mutagenesis pinpoint a ‘grab-and-stand’ binding model, wherein menthol interacts with residues in the S1–S4 voltage-sensor-like domain to shift channel voltage dependence toward physiological membrane potentials (Peier et al., 2002; Xu et al., 2020; Yin et al., 2018). TRPM8 opening allows Ca^2+^ entry and nerve depolarization, producing a cooling sensation even at ambient temperatures. At low to moderate menthol concentrations, TRPM8 mediates analgesia via central *κ*-opioid and group II/III mGluR pathways; at high concentrations, however, TRPM8 sensitization and off-target activation of TRPA1 can elicit cold allodynia and hyperalgesia (Li et al., 2022).

Behavioral assays in mice, including palpebral aperture measurements, blink frequency, and pawing responses following topical stimulation, provide a reproducible means of quantifying corneal sensory function. Mechanical corneal sensitivity was quantified using the Cochet–Bonnet esthesiometer, primarily reflecting mechano-nociceptor function, although polymodal afferents may also contribute (Belmonte et al., 2017). Chemical nociception was assessed using the Belmonte CO₂ esthesiometer. In parallel, chemosensory and thermosensory pathways were probed through topical application of capsaicin (TRPV1 agonist), hypertonic saline (a hyperosmolar stimulus primarily activating TRPV1-expressing polymodal nociceptors, with contributions from TRPM8-positive cold-sensitive afferents and other mechanosensitive pathways) (Bereiter et al., 2018; Li et al., 2019), and menthol (TRPM8 agonist), enabling targeted evaluation of polymodal and cold-sensitive afferent function. Tear volume measurements further assessed aqueous secretion. Structural characterization included OGD staining to evaluate epithelial barrier integrity and corneal whole-mount imaging to examine nerve density (McClellan et al., 2014). By integrating these behavioral and imaging readouts, it is possible to establish direct connections between epithelial barrier status, corneal innervation, neurosensory activity, and disease-relevant functional outcomes. Such integrative approaches are clinically relevant, as they may inform how distinct subtypes of DED, whether evaporative, aqueous-deficient, or injury-induced, differentiate in terms of symptom profile and therapeutic response.

In this study, we integrated multiple models (DS, LGE, aging, corneal debridement, and Mmp9 deficiency) with standardized behavioral and imaging assays to comprehensively evaluate corneal sensory function and epithelial barrier status. This approach addresses an underexplored need in the field by providing a comparative framework for how different environmental, surgical, and genetic insults alter corneal neuroepithelial function, thereby offering translational insights that could guide the design of targeted therapies for ocular surface disease.

Although individual models of dry eye disease and TRP channel function have been extensively studied, direct side-by-side evaluations across multiple paradigms remain scarce. In particular, how environmental stress (DS), chronic aqueous deficiency (LGE), aging, acute epithelial injury (corneal debridement), and genetic susceptibility (Mmp9 deficiency) differentially influence corneal sensory function and barrier integrity has not been systematically assessed. By integrating these diverse models with standardized behavioral imaging (palpebral aperture, blink, pawing), esthesiometry, tear volume assessment, and barrier assays, our study establishes a broad comparative framework for dissecting ocular surface pathophysiology across complementary disease contexts. This comparative strategy enables identification of both shared and model-specific alterations in TRPV1- and TRPM8-mediated responses, thereby contributing to a deeper understanding of the heterogeneity of clinical dry eye phenotypes and supporting the development of mechanism-based therapies.

## 2 Methods

### 2.1 Animals

All animal procedures complied with the ARVO Statement for the Use of Animals in Ophthalmic and Vision Research and were approved by the Institutional Animal Care and Use Committee at Baylor College of Medicine (protocol no. AN-8951). Female C57BL/6 J mice (Jackson Laboratories, Bar Harbor, ME) were used in two age groups: young (6–8 weeks) and aged (74–83 weeks). Female Mmp9KO (B6. FVB(Cg)-MMP9^tm1Tvu^/J, Jackson Laboratories) mice on a C57BL/6 background (8–10 weeks) were bred and maintained in our institutional animal facility. Detailed group numbers for each experiment are provided in Supplementary Table S1 and in individual figure legends.

Mice were housed under specific-pathogen-free conditions in individually ventilated cages (maximum of 5 animals per cage) with autoclaved bedding. Environmental parameters were maintained at 72–75 °F, 50–75% relative humidity, and a 12 h light/dark cycle. Animals had ad libitum access to standard rodent chow and sterilized water. Mmp9KO mice genotype was confirmed by PCR analysis.

### 2.2 Experimental groups

Mice were assigned to the following groups: B6 NS (non-stressed control) (*n* = 10), B6 DS5 (5-day desiccating stress) (*n* = 8–10), B6 aged (*n* = 10–12), B6 corneal debridement (CD) (*n* = 5), B6 lacrimal gland excision (LGE) (*n* = 8), B6 Sham Surgery (Sx) (*n* = 5), B6 LGE DS_LH_ (*n* = 8), and B6 Sham Sx DS_LH_ (*n* = 5). In addition, Mmp9KO mice were allocated to NS (*n* = 10), DS5 (*n* = 7–10), or CD (*n* = 5) groups (see Supplementary Table S1 for details).

#### 2.2.1 Desiccating stress

DS was induced as previously described (De Paiva et al., 2009; Yu et al., 2022). Tear secretion was inhibited by administering scopolamine hydrobromide (0.5 mg/mL; Greenpark, Houston) in the drinking water for 5 consecutive days (DS5). During this period, mice were housed in cages with a perforated plastic screen on one side to allow continuous lateral airflow from a fan positioned approximately 6 inches beside the cage, operating 16 h/day. Cages were placed inside a humidity- and temperature-controlled chamber (Darwin Chamber, St. Louis, MO) maintained at 75 °F and 25% relative humidity (RH). In selected experiments, mice were exposed to the same low-humidity airflow conditions without scopolamine to isolate the effects of environmental evaporative stress alone (DS_LH_). Food and water were provided ad libitum, and animals were monitored daily for weight loss and general activity. NS control mice were housed at 72–75 °F and 50–75% RH without exposure to air drafts.

#### 2.2.2 Lacrimal gland excision (LGE) and sham surgery

Extraorbital LGE was performed under 2–3% isoflurane anesthesia delivered via a nose cone, with depth of anesthesia confirmed by loss of pedal withdrawal reflex, as previously described (Mecum et al., 2020). Following aseptic preparation of the temporal periorbital region with povidone-iodine and 70% ethanol, a 2–3 mm skin incision was made at the junction between the ear root and the lateral canthus on the temporal side. The underlying connective tissue was gently separated by blunt dissection until the lacrimal gland became visible. The gland was carefully isolated from surrounding tissue and excised with minimal cautery to achieve hemostasis. The incision was closed with Dermabond Advanced Topical Skin Adhesive (Ethicon, Somerville, NJ, USA).

Sham Sx controls underwent identical surgical exposure and gland visualization without gland excision. Postoperative analgesia consisted of subcutaneous meloxicam (5 mg/kg, once daily for 3 days). Animals were monitored daily for signs of pain or distress and were housed individually in sterile cages with ad libitum access to food and water until fully recovered.

#### 2.2.3 Corneal debridement

Corneal epithelial debridement was performed as previously described, with minor modifications (Abu-Romman et al., 2024). Bilateral epithelial removal was carried out under general anesthesia with 2–3% isoflurane delivered via a nose cone, with adequate depth confirmed by loss of pedal withdrawal reflex. After induction of anesthesia, a 2 μL drop of 0.5% proparacaine hydrochloride was instilled onto the ocular surface and excess fluid gently blotted with filter paper. The central cornea was gently marked with a sterile 1.5-mm trephine (McKesson Argent Surgical Systems, Richmond, VA, USA) under a dissecting microscope. The epithelial layer within the marked area was then carefully removed by gentle scraping with a sterile disposable rounded epithelial spatula (Corzamedical no. 5I-0003, Parsippany, NJ).

For perioperative analgesia, mice were provided with a medicated diet containing carprofen (2 mg/tablet, Bio-Serv, SMD 150–2; one tablet per animal daily), starting 24 h before the procedure and maintained until 48 h afterward. No topical agents, including antibiotics or lubricants, were administered during the recovery period. All mice were monitored regularly after surgery to monitor healing and ensure the absence of pain or distress.

### 2.3 Corneal sensitivity measurement

#### 2.3.1 Corneal mechanical sensitivity-Cochet–Bonnet esthesiometer

Mechanical sensitivity was assessed with a Cochet–Bonnet esthesiometer (Luneau Ophthalmologie, Chartres Cedex, France) as previously described (Galletti et al., 2023a). Mice were gently restrained without anesthesia, and measurements were obtained at the central cornea under constant room illumination. The monofilament length was initially set at 60 mm and decreased in 5 mm steps (60 → 0 mm) until a positive response (complete blink and/or eye retraction) was observed. For each length, the filament was applied perpendicularly and bend slightly upon contact for ~1 s. Three trials were performed with an inter-trial interval of ≥10 s. The threshold was defined as the longest length that elicited a positive response in ≥2 of 3 trials. Because corneal sensitivity can change depending on the time of the day, all measurements were performed at 10:00 a.m. ± 20 min by the same experimenters.

#### 2.3.2 Corneal chemical sensitivity-Belmonte CO₂ esthesiometer

Chemical sensitivity was evaluated using a Belmonte CO₂ esthesiometer, which delivers controlled pulses of humidified air mixed with defined concentrations of CO₂ to the ocular surface. Mice were gently restrained without anesthesia, and stimuli were directed perpendicularly to the central cornea from a fixed distance of 3 mm. Pulses were delivered at increasing CO₂ concentrations in stepwise increments (e.g., 10, 20, 30%), each lasting ~2 s with ≥30 s between pulses to prevent sensitization. Stimuli were delivered at a controlled flow rate of 20 mL/min at a temperature of 22 °C, using regulated CO₂ and air pressures (CO₂: 25 psi working pressure from a 2,500 psi source; air: 4 psi from a 1,000 psi source), ensuring stable and reproducible stimulus delivery across experiments. A positive response was defined as a complete blink and/or retraction of the globe into the ocular orbit within 2 s of stimulus onset. The chemical threshold was defined as the lowest CO₂ concentration that elicited a positive response in ≥2 of 3 trials. Each eye was tested in triplicate, and the median threshold was used for analysis. Because corneal sensitivity can vary depending on the time of day, all measurements were performed at 1:00 p.m. ± 20 min by the same experimenters.

### 2.4 Corneal barrier function

Corneal epithelial barrier integrity was assessed using Oregon Green 488–dextran (OGD, 70 kDa; Invitrogen, Eugene, OR) prepared at 5 mg/mL in sterile PBS. A 1.0 μL drop of OGD was applied bilaterally to the corneal surface using a calibrated micropipette, avoiding direct contact with the ocular surface. Following application, mice were placed in a dark environment for 1 min to allow dye uptake. Each eye was then rinsed with 2 mL of Balanced Salt Solution to remove residual fluorophore. Animals were then euthanized by overdose of isoflurane anesthesia followed by cervical dislocation as a secondary method to ensure death, and corneal fluorescence images were acquired post-mortem. This protocol was performed in all experimental groups under identical conditions.

Fluorescence images were obtained using a fluorescence stereomicroscope equipped with a GFP filter set (Ex 488 nm, Em 510–550 nm). Identical imaging parameters (exposure time, gain, gamma) were applied across all animals. Image analysis was performed in NIS-elements using a fixed-size circular region of interest (ROI) centered on the corneal apex. Background intensity was sampled from adjacent sclera and subtracted from corneal measurements. The average from both eyes of each animal was used for statistical analysis.

### 2.5 Tear volume

Tear secretion was measured using a custom-prepared phenol red thread, generated in our laboratory following previously published murine protocols for phenol red thread testing (Puentes et al., 2024). Mice were gently restrained without anesthesia, and all procedures were performed under constant room illumination. The folded end of the thread was placed at the lateral lower eyelid margin, close to the lateral canthus, without touching the cornea, and left in place for 30 s. The length of the wetted (color-changed) portion was recorded in millimeters from the fold to the transition point. Each eye was tested once, and the average value of both eyes was used for statistical analysis. To minimize circadian variability, all measurements were conducted at 11:00 a.m. ± 20 min by the same experimenters.

### 2.6 Video imaging of palpebral aperture and blink/pawing responses

Palpebral aperture dimensions and blink/pawing responses were quantified using a custom video imaging system. Mice were gently restrained in a 50 mL conical tube that allowed free head movement and unobstructed visualization of the eyes. Recordings were obtained with an Alvium 1800 U-511 m monochrome camera (Allied Vision, Stadtroda, Germany) equipped with a 25 mm C-Series fixed focal length lens (aperture f/1.4), positioned at a constant, predetermined distance from the animal. Videos were captured at 60 frames per second for 2 min using StreamPix 9 software (NorPix, Montreal, Canada). To reduce background artifacts and improve image stability, a black cardboard panel was placed behind the mice during recordings. All experiments were conducted in the same room and at the same recording station to ensure consistent background and lighting conditions.

Each mouse was first recorded without stimulation to establish a baseline (Supplementary Video S1). For animals that underwent LGE, Sham Sx, DS5, or CD, an additional recording was performed after recovery from the procedure but before any topical stimulus. Palpebral aperture was measured from digital video frames at baseline and post-stimulus. At least 10 evenly spaced frames per minute were analyzed per recording. Blink frequency (complete lid closure) and pawing responses (forepaw contacts to the periocular region) were manually counted from the same video segments (Supplementary Video S2). To minimize circadian variability, all recordings were performed at approximately the same time in the day by the same experimenters.

### 2.7 Palpebral aperture and blink measurement in humans

In human participants, palpebral aperture height-to-width ratio (termed eye aspect ratio - EAR) and spontaneous blink frequency were quantified from 2-min video recordings acquired under baseline conditions (Supplementary Video S3) and during a controlled evaporative challenge using a fan. All participants provided written informed consent prior to participation, and all procedures were conducted in accordance with a protocol approved by the Baylor College of Medicine Institutional Review Board (IRB).

Participants were categorized as either normal controls or patients with clinically diagnosed DED. DED was defined by a tear film breakup time (TBUT) < or equal to 8 s in conjunction with significant symptom burden, as determined by SANDE questionnaire scores greater than 40. Demographic and clinical characteristics of the control and DED participants, including age, sex, TBUT, SANDE scores, blink rate, and EAR under baseline and evaporative stress conditions, are summarized in Supplementary Table S2. Video recordings were processed using a fully automated, custom-developed analysis pipeline that extracted eyelid aperture dynamics and blink events on a frame-by-frame basis. The software identified both partial blinks, defined as ≥50% eyelid closure, and complete blinks, allowing comprehensive characterization of blink behavior. These quantitative metrics were used to assess eyelid behavior and ocular sensory function under both physiological conditions and evaporative stress.

The DED cohort encompassed a range of clinically distinct subtypes, including aqueous-deficient dry eye associated with Sjögren’s syndrome (ATD-SS, *n* = 7), non-Sjögren aqueous-deficient dry eye (ATD, *n* = 2), mixed DED (*n* = 19), conjunctivochalasis (CCh, *n* = 2), meibomian gland dysfunction (MGD, *n* = 5).

### 2.8 Response to capsaicin, hypertonic saline, and menthol

Corneal chemosensory responses were evaluated by applying topical TRP channel agonists, including capsaicin (0.33 μM), hypertonic saline (HS, 0.4 M), and menthol (20 μM). Drug concentrations were selected based on preliminary titration experiments conducted in an independent dataset (data not shown). Stimuli were applied as 2 μL drops to the central cornea using a calibrated micropipette, taking care to avoid direct contact with the ocular surface. Each agonist was tested on separate experimental days to prevent sensitization or cross-desensitization effects.

Prior to each stimulus, a baseline recording was obtained under identical conditions. Behavioral responses (palpebral aperture, blink frequency, and pawing counts) were recorded and analyzed as described above. Measurements were performed across all experimental groups, including NS controls, DS5, LGE, Sham Sx, Aged, Mmp9KO and CD mice.

### 2.9 Image analysis

Video recordings were processed using StreamPix 9 software (NorPix, Montreal, Canada), which enabled frame-by-frame playback and export. To standardize analysis across groups, one frame was selected for every 100 frames. If the designated frame was of poor quality (e.g., blurred by motion), the closest acceptable frame (±10 frames) was selected instead.

Palpebral aperture dimensions were quantified manually using ImageJ (NIH, Bethesda, MD, USA). For each selected frame, both eyelid width (horizontal palpebral fissure) and height (vertical distance between upper and lower eyelids) were measured in pixels. To correct for potential variations in recording distance, aperture size was expressed as the height-to-width ratio (HWR), providing a normalized dimensionless index for comparisons across animals and groups.

Blink responses were manually counted by reviewing the full video playback in StreamPix, and pawing events were counted in the same manner.

### 2.10 Statistics

All statistical analyses were performed using GraphPad Prism (Version 10.3.1). For pairwise comparisons, the nonparametric Mann–Whitney U test was used. For comparisons across involving more than two groups, the Kruskal–Wallis test was applied. Data are presented as mean ± SD, and the unit of analysis was *n = animals* (with values averaged per animal when both eyes were measured). Statistical significance was defined as *p* < 0.05.

It should be noted that comparisons were performed within independent experimental cohorts. For example, the B6 young vs. aged groups and the B6 NS vs. MMP-9 KO NS groups were assessed in separate sets of mice. Because corneal mechanical sensitivity and related measures can vary depending on time of day, environmental conditions, and facility-specific factors, results are only directly comparable within each cohort. Consequently, mean ± SD values for the B6 NS group may appear different across figures or text descriptions, as they represent distinct experimental cohorts rather than a single pooled control population.

## 3 Results

### 3.1 Impact of dry eye on corneal barrier function and sensitivity

#### 3.1.1 Tear volume

In the B6 strain, tear secretion was markedly reduced under acute and chronic dry eye conditions. In the DS5 group, tear volume was below the detection limit of the phenol red thread test (0.00 ± 0.00 mm), representing a profound reduction compared with B6 NS controls (3.53 ± 1.07 mm, *p* < 0.0001). LGE (0.76 ± 1.07 mm at 2 weeks; 1.40 ± 1.52 mm at 3 weeks) also significantly reduced tear output relative to sham-operated mice at both 2 weeks (3.69 ± 1.11 mm, *p* = 0.0002) and 3 weeks (4.50 ± 1.73 mm, *p* = 0.015), and at 2 weeks also differed significantly from B6 NS controls (*p* = 0.0003). Sham Sx alone did not alter tear production (*p* = 0.86). When LGE was combined with DS5 (0.00 ± 0.00 mm), tear secretion approached zero and differed significantly from B6 NS (*p* = 0.0001). A summary of tear volume measurements across all models is provided in Table 1.

**Table 1:** Directional changes in tear secretion, corneal sensitivity, and nerve density across experimental groups.

	MMP-9 KO	LGE	Sham Sx	DS5				B6 Aged
				B6	MMP-9 KO	LGE	Sham Sx	
Tear volume	≈	↓↓↓	≈	↓↓↓↓	↓	↓↓↓↓	≈	↑↑
Corneal mechanical sensitivity	↓↓	↓↓↓↓	↓↓↓	↓↓↓↓	↓↓	↓↓↓↓	↓↓↓	↓
Corneal CO2 gas sensitivity	↓	≈	≈	≈	↓↓	≈	≈	≈
Corneal nerve density		_≈_^12^	_≈_^12^	↓^42^				↓^42^

Directional arrows indicate the magnitude and direction of change relative to the corresponding non-stressed B6 control group: ↓/↑ = *p* < 0.05, ↓↓/↑↑ = *p* < 0.01, ↓↓↓/↑↑↑ = *p* < 0.001, ↓↓↓↓/↑↑↑↑ = *p* < 0.0001, ≈ = no significant difference.

NS, Non stressed; DS, Desiccating stress; LGE, Lacrimal gland excision; Sham Sx, Sham surgery.

Importantly, Sham Sx DS_LH_ mice (4.00 ± 0.61 mm), which were exposed to low humidity without scopolamine, showed no significant change in tear volume compared with either Sham Sx controls (*p* = 0.71) or B6 NS controls (*p* = 0.68), indicating that low humidity alone does not suppress aqueous tear secretion.

Before examining the effects of aging on tear secretion, it is important to note that aging is a well-established risk factor for ocular surface dysfunction. Prior studies have shown that aged B6 mice exhibit multiple dry eye-like features, including impaired epithelial barrier integrity, reduced goblet cell density, meibomian gland abnormalities, and lymphocytic infiltration within the lacrimal gland, despite often demonstrating increased aqueous tear output relative to young controls (Bron et al., 2017; Galletti et al., 2023b). This distinction between tear quantity and tear film composition provides critical context for interpreting age-related tear volume differences. In this context, aged B6 mice (5.75 ± 1.91 mm) exhibited significantly higher tear volume than young B6 controls (*p* = 0.02), consistent with increased aqueous output despite age-associated tear film dysfunction.

At baseline, Mmp9KO NS (4.90 ± 2.73 mm) did not differ from B6 NS controls (*p* = 0.48). Following DS, Mmp9KO DS5 mice (1.25 ± 0.9 mm) showed a significant reduction in tear volume compared with both Mmp9KO NS (*p* = 0.0007) and B6 NS (*p* = 0.02), demonstrating that Mmp9 deficiency does not prevent DS-induced tear loss.

Together, these results show that both acute and chronic dry eye conditions markedly reduce tear secretion and volume, whereas aging increases tear output, and Mmp9 deficiency does not confer protection against tear loss in the DS model. The lack of tear reduction in Sham Sx DS_LH_ confirms that scopolamine is required to induce tear deficiency under low-humidity conditions.

#### 3.1.2 Corneal barrier is disrupted in dry eye and aging

**Figure 1:**
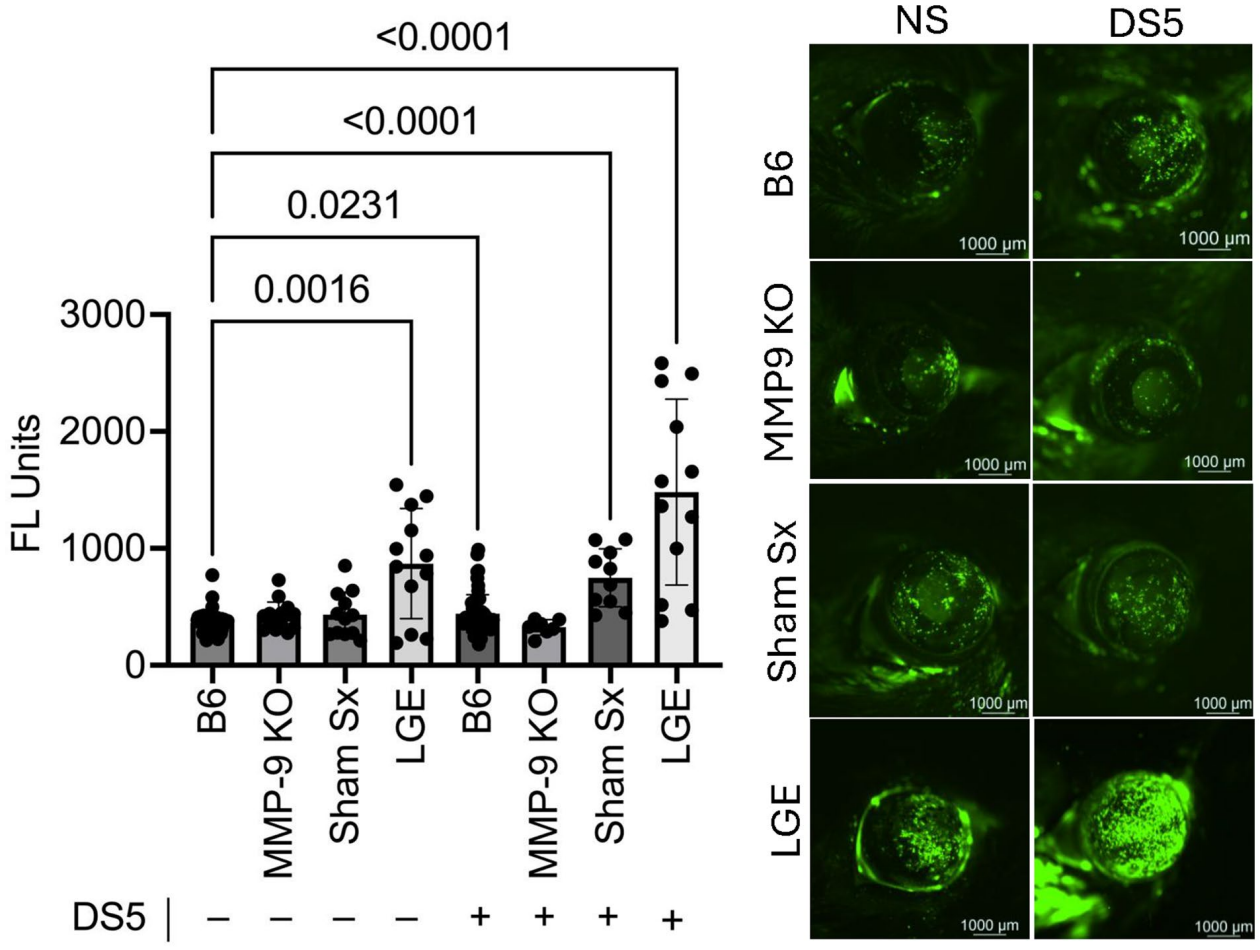
Corneal epithelial barrier disruption across dry eye models assessed by Oregon Green dextran (OGD) staining. **(Left)** Quantification of corneal OGD fluorescence intensity in B6 and Mmp9KO mice under non stressed (NS) and desiccating stress for 5 days (DS5) conditions, as well as in chronic aqueous-deficient dry eye (lacrimal gland excision) and sham-operated controls. Data are presented as mean ± SD. Each dot represents an individual eye. All groups were compared to the B6 NS unless otherwise specified. *p* values < 0.05 were considered statistically significant. *p*-values displayed above brackets indicate pairwise comparisons between the groups connected by each bracket. Statistical analysis was performed using the Kruskal–Wallis test with Dunn’s multiple comparisons. **(Right)** Representative corneal surface OGD fluorescence images from each experimental group under NS and DS5 conditions. Increased punctate and confluent epithelial fluorescence indicates impaired corneal epithelial barrier integrity. Scale bar = 1,000 μm.

Acute environmental stress caused a clear breakdown of the epithelial barrier (Figure 1). B6 DS5 mice showed significantly elevated OGD fluorescence compared with B6 NS controls (441.4 ± 161.8 vs. 361.7 ± 85.9 gray levels, *p* = 0.02), indicating increased corneal permeability following five days of low humidity and anticholinergic suppression of tear output.

LGE (870.0 ± 470.0 gray levels) resulted in a substantial increase in OGD intensity compared with B6 controls (*p* = 0.0016) and also showed significantly higher permeability than sham-operated mice (433.3 ± 193.6 gray levels, *p* = 0.045), confirming that chronic aqueous deficiency alone is sufficient to impair barrier integrity. As expected for a chronic dry-eye model, LGE mice displayed elevated OGD fluorescence even under normal humidity conditions.

When LGE was combined with DS (LGE DS_LH_), corneal barrier breakdown was markedly exacerbated. In the post-surgical low-humidity paradigm without scopolamine, OGD values in LGE DS_LH_ mice were significantly higher than in Sham Sx DS_LH_ animals (1,481 ± 795.4 vs. 749.4 ± 247.8 gray levels, *p* < 0.0001), indicating that pre-existing lacrimal gland loss markedly amplifies the barrier-disruptive effects of environmental stress.

Sham Sx mice under normal humidity showed no difference relative to B6 controls (*p* > 0.9999). However, when Sham Sx animals were exposed to low humidity without scopolamine (Sham Sx DS_LH_), OGD staining increased compared with B6 NS (*p* < 0.0001), indicating that environmental stress alone can promote barrier compromise even in the absence of pharmacologic tear suppression. Nevertheless, the significantly higher OGD intensities in LGE DS_LH_ vs. Sham Sx DS_LH_ (*p* = 0.036) demonstrate that chronic aqueous deficiency further exacerbates low-humidity–induced barrier disruption.

Although aging was not directly assessed in this dataset, prior studies have reported increased corneal permeability with age (McClellan et al., 2014), supporting the use of this model.

Production of the inflammatory protease, MMP-9 which disrupts the corneal epithelial barrier, increases in response to DS in B6, but not in the Mmp9KO strain. Representative images of MMP-9 immunostaining in corneas of these strains are shown in Supplementary Figure S1. Under NS conditions, Mmp9KO mice (418.1 ± 124.0 gray levels) showed OGD staining comparable to B6 NS controls (*p* = 0.86), indicating preserved baseline epithelial integrity in the absence of MMP-9. Importantly, exposure to desiccating stress did not increase corneal permeability in Mmp9KO mice. OGD intensities in Mmp9KO DS5 animals (325.3 ± 65.45 gray levels) were not significantly different from Mmp9KO NS (*p* > 0.09) or B6 NS (*p* > 0.99), but were significantly lower than in B6 DS5 mice (441.0 ± 161.0 gray levels; *p* = 0.013). These results indicate that Mmp9 deficiency protects against the DS-induced increase in epithelial permeability observed in wild-type mice, preserving barrier function at levels comparable to NS controls.

Across models, both acute environmental stress (DS5) and chronic tear deficiency (LGE) significantly compromised corneal barrier integrity, while aging produced a milder but measurable increase in epithelial permeability. In contrast, Mmp9 deficiency mitigated DS-induced barrier loss, highlighting a potential protective role against environmentally driven epithelial damage.

#### 3.1.3 Corneal sensory function across dry eye models

##### 3.1.3.1 Mechanical sensitivity (Cochet–Bonnet esthesiometry)

Mechanical corneal sensitivity, primarily reflecting mechano-nociceptor function, decreased across both acute and chronic dry eye models (Supplementary Figure S2A; Table 1). B6 mice exposed to DS5 showed a marked reduction in mechanical threshold compared with B6 NS controls (2.30 ± 0.47 vs. 5.77 ± 0.48 cm, *p* < 0.0001).

Chronic aqueous deficiency produced a similarly profound effect. LGE mice demonstrated significantly lower thresholds (2.16 ± 1.44 cm) than both B6 NS (*p* < 0.0001) and Sham Sx NS controls (*p* = 0.0015), confirming that lacrimal gland removal alone is sufficient to impair mechanoreceptor function. Mechanical sensitivity also declined modestly following Sham Sx (3.69 ± 0.91 cm, *p* = 0.0005).

Under DS, both LGE DS_LH_ (2.16 ± 0.69 cm) and Sham Sx DS_LH_ (3.10 ± 0.63 cm) mice displayed reduced mechanical sensitivity compared with B6 NS (*p* < 0.0001 and *p* = 0.0006, respectively). However, mechanical thresholds did not differ between LGE DS_LH_ and Sham Sx DS_LH_ mice (*p* = 0.15), nor between LGE and LGE DS_LH_ groups (*p* = 0.59), indicating that DS_LH_ exposure does not further reduce mechanical sensitivity beyond the deficit induced by lacrimal gland removal alone.

Aged B6 mice exhibited significantly reduced mechanical corneal sensitivity compared with young controls (4.31 ± 1.11 vs. 5.77 ± 0.48 cm, *p* = 0.02). This decrease aligns with our prior observations that aging is accompanied by diminished corneal tactile responsiveness and progressive rarefaction of subbasal nerves, suggesting an intrinsic age-related decline in mechanosensory function independent of tear volume or surface inflammation (Abu-Romman et al., 2024; Stepp et al., 2018).

Mmp9 deficiency altered the magnitude of DS-induced sensitivity loss. Although Mmp9KO NS mice (4.18 ± 1.56 cm) showed mildly reduced thresholds compared with B6 NS (*p* = 0.004), mechanical thresholds did not differ between KO NS and KO DS5 (3.90 ± 1.41 cm, *p* = 0.30), indicating that desiccating stress did not further diminish mechanoreceptor function in Mmp9–deficient mice. Importantly, the decline following DS5 was substantially smaller in Mmp9KO mice than in WT DS5 mice (3.90 ± 1.41 vs. 2.30 ± 0.47 cm, *p* = 0.002), demonstrating partial preservation of mechanical sensitivity in the absence of *Mmp9*.

##### 3.1.3.2 Chemical sensitivity (CO₂ esthesiometry)

Chemical corneal sensitivity exhibited a distinct pattern from mechanical sensitivity, with acute desiccating stress producing the largest decrement (Supplementary Figure S2B; Table 1). DS5-exposed B6 showed a reduction in CO₂ sensitivity compared with B6 NS controls (37.86 ± 19.68 vs. 26.67 ± 34.47 mL/min) that did not reach statistical significance (*p* = 0.0508).

Chronic aqueous deficiency produced a smaller but still detectable shift. LGE mice (18.00 ± 13.04 mL/min) showed significantly increased CO₂ sensitivity compared with sham-operated animals (45.00 ± 33.91 mL/min, *p* = 0.0009), consistent with heightened chemosensory responsiveness following chronic lacrimal gland removal. However, the difference between LGE and B6 NS mice was not statistically significant (*p* = 0.87), reflecting high variability in baseline responses. Sham Sx alone did not alter CO₂ sensitivity relative to B6 NS (*p* = 0.16).

Environmental stress had differential effects depending on baseline tear status. When exposed to low humidity, LGE DS_LH_ mice showed the lowest CO₂ sensitivity, markedly reduced compared with Sham Sx DS_LH_ mice (10.83 ± 14.29 vs. 28.00 ± 40.71 mL/min, *p* = <0.0001). This indicates that chronic tear deficiency amplifies the chemosensory impact of environmental stress, whereas low humidity alone has a weaker effect when lacrimal gland function is intact.

Aging produced a pattern different from that observed above. Aged B6 mice (11.25 ± 3.54 mL/min, *p* = 0.34) showed CO₂ responses comparable to young controls, suggesting that polymodal nociceptor function remains largely preserved with age despite known age-related epithelial changes.

Genotype exerted a strong influence on chemical sensitivity. Mmp9KO mice consistently exhibited reduced CO₂ responsiveness relative to wild-type animals, reflected by markedly higher CO₂ thresholds across conditions. KO NS mice (55.00 ± 39.89 mL/min) showed substantially lowered chemical sensitivity compared with B6 NS controls (26.67 ± 34.47 mL/min, *p* = 0.035). Following DS, KO DS5 animals demonstrated the highest thresholds observed in any group (70.00 ± 27.08 mL/min), remaining significantly less responsive than B6 NS mice (*p* = 0.004). Mmp9KO DS5 mice exhibited significantly higher CO₂ thresholds than B6 DS5 animals (*p* = 0.01), indicating a greater impairment of polymodal nociceptor signaling in the absence of MMP-9. Notably, CO₂ thresholds in KO mice changed little with DS (KO NS vs. KO DS5, *p* = 0.41), indicating that the absence of MMP-9 establishes a chronically blunted chemosensory state and prevents the stress-induced enhancement in CO₂ sensitivity observed in wild-type mice.

Taken together, these results show that CO₂ chemosensation is most strongly affected by acute environmental stress, more modestly influenced by chronic tear deficiency, preserved with aging, and remodeled in a distinct way by Mmp9 deficiency. This pattern differs from mechanical sensitivity and highlights the modality-specific vulnerability of corneal nociceptive pathways across dry eye states.

### 3.2 Dry eye reduces palpebral aperture

#### 3.2.1 Dry eye models

**Figure 2:**
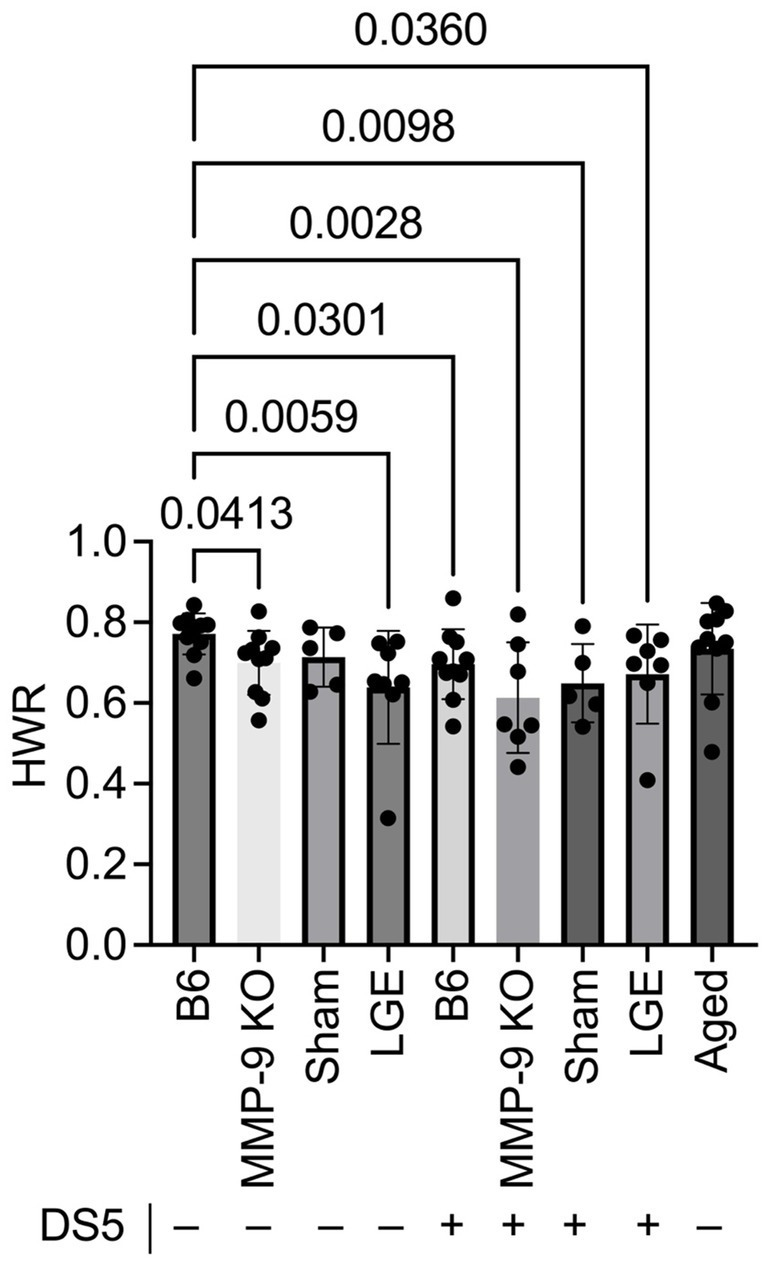
Palpebral aperture (HWR) in B6 NS mice compared with dry eye models. Palpebral aperture, expressed as the height-to-width ratio (HWR), was measured in B6 non stressed (NS) mice and in dry eye models including sham surgery, lacrimal gland excision, desiccating stress for 5 days (DS5), and aging, as indicated. Data are shown as mean ± SD, with each dot representing one eye. All groups were compared to the B6 NS reference group unless otherwise indicated. *p* values < 0.05 were considered statistically significant. *p*-values displayed above brackets indicate pairwise comparisons between the groups connected by each bracket. Statistical analysis was performed using the Kruskal–Wallis test followed by Dunn’s *post hoc* test without correction for multiple comparisons.

Acute environmental stress induced consistent narrowing of the palpebral aperture (Figure 2). B6 DS5 mice (0.70 ± 0.09) showed significantly reduced aperture compared with B6 NS controls (0.77 ± 0.05, *p* = 0.03), demonstrating that 5 days of low-humidity exposure is sufficient to decrease eyelid opening.

Chronic aqueous deficiency produced a stronger phenotype. LGE NS mice (0.64 ± 0.14) showed significantly reduced aperture compared with B6 NS (*p* = 0.006), confirming that loss of lacrimal gland function alone is sufficient to narrow the eyelid opening. LGE DS_LH_ mice (0.67 ± 0.12) also differed significantly from B6 NS controls (*p* = 0.036), but aperture narrowing in LGE DS_LH_ did not exceed that of LGE NS, suggesting a plateau effect rather than additive impairment (*p* = 0.4).

Sham manipulation itself did not alter baseline aperture, as Sham Sx NS mice (0.71 ± 0.07) did not differ significantly from B6 NS (*p* = 0.19). However, Sham Sx DS_LH_ mice (0.65 ± 0.10) exhibited significant narrowing relative to B6 NS (*p* = 0.0098), indicating that environmental stress alone can modestly reduce aperture even without pharmacologic tear suppression.

Aged mice (0.73 ± 0.11) showed no significant difference compared with B6 NS (*p* = 0.44), indicating that aging alone does not appreciably affect palpebral aperture.

Reduced baseline aperture was noted in the *Mmp9* gene–deleted strains, as KO NS exhibited significantly lower baseline aperture (0.70 ± 0.08) compared with B6 NS controls (*p* = 0.04). Following DS5, Mmp9KO showed further significant aperture narrowing (0.61 ± 0.14) relative to B6 NS controls (*p* = 0.003). Although aperture decreased from KO NS to KO DS5, this within-genotype change did not reach statistical significance.

Blink frequency remained low across baseline groups, including B6 NS (0.10 ± 0.32 blinks/min), Sham Sx NS (1.00 ± 1.41 blinks/min), LGE NS (0.25 ± 0.71 blinks/min), Mmp9KO NS (0.20 ± 0.42 blinks/min), and aged mice (0.00 blinks/min), with no significant differences among them. Following DS, blink rates remained unchanged in B6 DS5 (0.00 blinks/min) and Mmp9KO DS5 (0.00 blinks/min). In contrast, Sham Sx DS_LH_ (1.20 ± 1.30 blinks/min, *p* = 0.02) and LGE DS_LH_ (1.43 ± 1.81 blinks/min, *p* = 0.009) showed significantly increased blink responses relative to B6 NS, suggesting mild ocular surface irritation when surgical manipulation or tear deficiency was combined with environmental stress. No differences were detected between B6 NS and aged mice (*p* = 0.60). Pawing behavior was absent across all baseline groups and did not change significantly under any experimental condition.

#### 3.2.2 Dry eye patients

Human palpebral aperture measurements demonstrated reduced eyelid opening in patients with dry eye disease compared with healthy individuals. Under normal conditions without airflow, patients with dry eye showed significantly lower EAR values (0.25 ± 0.04) than controls (0.29 ± 0.04, *p* = 0.002), indicating a baseline reduction in eyelid opening height consistent with increased eyelid narrowing. This difference persisted when a directed airflow stimulus was applied. EAR remained significantly lower in patients (0.25 ± 0.05) than in controls (0.27 ± 0.04, *p* = 0.026). These findings parallel the aperture narrowing observed in acute and chronic mouse dry eye models, suggesting that eyelid opening responses to ocular surface stress are conserved across species.

**Figure 3:**
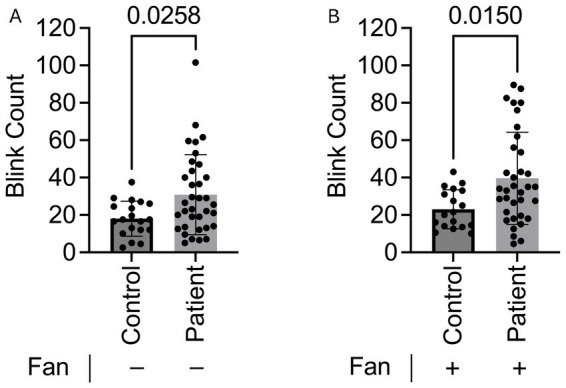
Blink frequency in humans under baseline and evaporative stress conditions. Blink frequency was measured in control subjects and dry eye disease patients under baseline conditions [**(A)** fan off] and during evaporative stress induced by a fan **(B)**. Data are shown as mean ± SD, with each dot representing an individual subject. *p*-values displayed above brackets indicate pairwise comparisons between control and patient groups within each condition. *p* values < 0.05 were considered statistically significant. Statistical analysis was performed using the Mann–Whitney U test.

Human blink frequency was significantly elevated in dry eye patients compared with healthy controls. Under normal conditions without airflow, dry eye patients exhibited higher blink rates (30.84 ± 21.42 blinks/1 min) relative to controls (18.00 ± 9.37 blinks/1 min), with a significant difference between groups (*p* = 0.026) (Figure 3A). This pattern persisted when a directed airflow stimulus was applied, blink frequency remained significantly higher in patients (39.60 ± 24.66 blinks/1 min) compared with controls (23.06 ± 10.41 blinks/1 min, *p* = 0.015) (Figure 3B). These findings mirror the modest blink increases observed in select mouse dry eye models (Sham Sx DS_LH_ and LGE DS_LH_) and suggest that humans with dry eye exhibit heightened blink activity both at baseline and when exposed to airflow.

### 3.3 Response to chemical stimulants in dry eye

#### 3.3.1 Capsaicin

**Figure 4:**
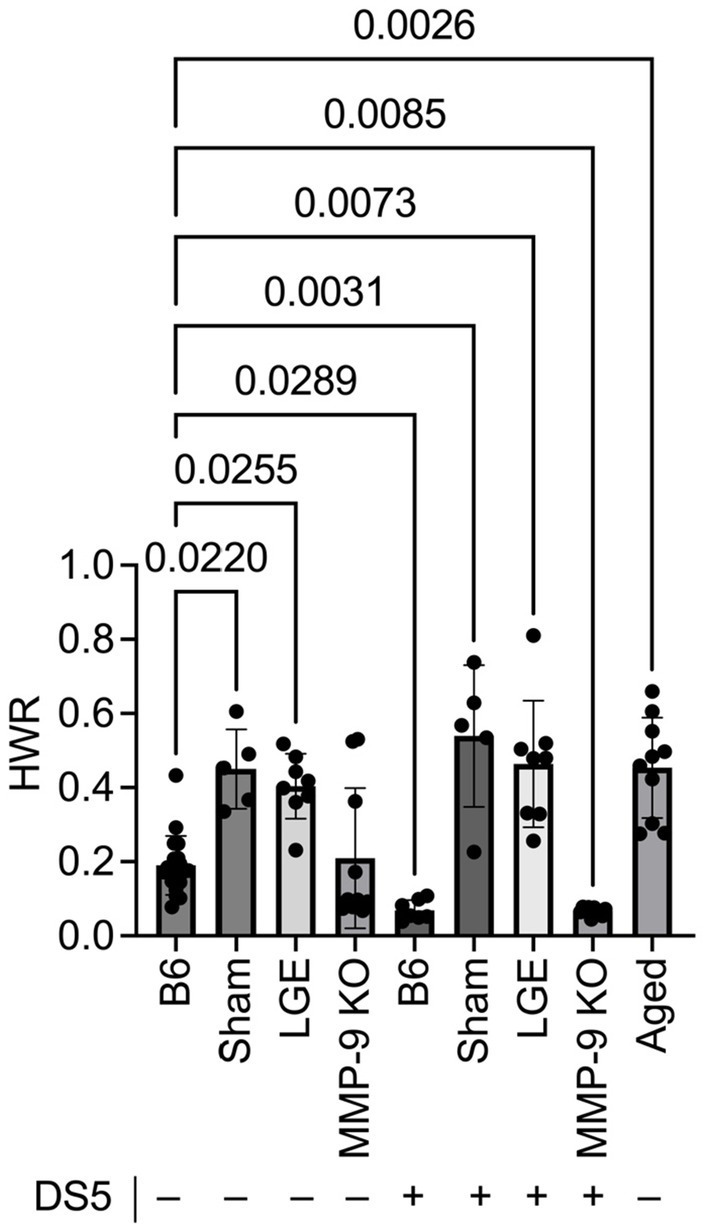
TRPV1-mediated sensory responses relative to capsaicin-treated B6 NS. Palpebral aperture (HWR) following topical capsaicin application was measured in B6 and Mmp9KO mice under non-stressed (NS) and desiccating stress for 5 days (DS5) conditions, as well as in sham-operated, lacrimal gland excision, and aged groups, as indicated. Data are shown as mean ± SD, with each dot representing one eye. All comparisons were made following capsaicin application to the B6 NS reference group unless otherwise indicated. *p* values < 0.05 were considered statistically significant. *p*-values displayed above brackets indicate pairwise comparisons between the groups connected by each bracket. Statistical analysis was performed using the Kruskal–Wallis test followed by Dunn’s post hoc test without correction for multiple comparisons.

Application of the TRPV1 agonist capsaicin produces model-specific alterations in eyelid aperture and nocifensive behaviors (Figure 4). Compared with capsaicin-treated B6 NS controls (0.19 ± 0.08), the B6 DS5 group showed a significant reduction in palpebral aperture (0.07 ± 0.03, *p* = 0.03), indicating that desiccating stress enhances TRPV1-evoked eyelid closure.

The LGE NS group showed statistically significant attenuation in capsaicin-evoked response (0.40 ± 0.09) compared with B6 NS + capsaicin controls (*p* = 0.02). A similar reduction was observed in the Sham Sx NS group (0.45 ± 0.11, *p* = 0.02), indicating that surgical manipulation alone may influence TRPV1-mediated eyelid responses. Exposure to low humidity further reduced TRPV1-mediated eyelid closure in LGE DS_LH_ mice (0.46 ± 0.17, *p* = 0.007), suggesting that environmental stress does not restore TRPV1 responsiveness and instead further suppresses it in the context of chronic tear deficiency. Similarly, the Sham Sx DS_LH_ group (0.54 ± 0.19) exhibited a significantly weaker capsaicin-evoked narrowing relative to B6 NS controls (*p* = 0.003).

Aged mice (0.45 ± 0.13) also show a significantly *reduced* capsaicin-evoked eyelid-closure response compared with B6 NS (*p* = 0.003), indicating aging blunts rather than enhances TRPV1 sensitivity.

Mmp9 deficiency produces a distinct phenotype. Mmp9KO NS + capsaicin (0.21 ± 0.19) does not differ from B6 NS + capsaicin (*p* = 0.86), yet Mmp9KO DS5 (0.07 ± 0.01) demonstrated significantly stronger eyelid narrowing (*p* = 0.008), indicating heightened TRPV1 sensitivity under DS.

Topical capsaicin elicits robust blink activity in B6 (20.50 ± 13.80 blinks/min). This response is markedly reduced under acute DS, as B6 DS5 have far fewer blinks (3.13 ± 5.72 blinks/min, *p* < 0.0001), consistent with DS-associated suppression of TRPV1-mediated eyelid reflexes.

Chronic tear deficiency produces an intermediate phenotype. Topical capsaicin induces moderate blink activity (8.75 ± 4.27 blinks/min) following LGE, and the level is comparable when LGE is combined with DS (13.38 ± 5.90 blinks/min, *p* = 0.22). Sham-operated animals behave similarly. Following Sham Sx, blink rate is like LGE (11.00 ± 4.90 blinks/min), whereas Sham Sx DS_LH_ show slightly lower responses (5.00 ± 3.00 blinks/min). Together, these findings indicate that chronic aqueous deficiency and surgical manipulation attenuate capsaicin-evoked blinking but do not eliminate the response entirely.

Aged mice display low blink frequencies (4.70 ± 2.75 blinks/min), significantly lower than B6 NS (*p* = 0.003), indicating reduced TRPV1-driven blink reflexes with aging.

*Mmp9* gene deletion produces a distinct pattern. Mmp9KO NS exhibit lower blink activity (11.20 ± 9.94 blinks/min) than WT controls, and KO DS5 reduces the responses (6.50 ± 2.99 blinks/min). This indicates that *Mmp9* deficiency constrains both baseline and stress-evoked blink responsiveness to capsaicin.

Capsaicin-evoked pawing responses shows a distinct pattern compared with eyelid closure and blinking. B6 NS (7.30 ± 5.93 pawing/min) exhibited robust pain-related forepaw activity, whereas Sham Sx, LGE, Sham Sx DS_LH_, LGE DS_LH_, and aged mice show no pawing (all means = 0), with significantly lower responses than B6 NS (*p* = 0.008, *p* = 0.002, *p* = 0.008, *p* = 0.002, respectively). B6 DS5 mice (0.38 ± 0.52 pawing/min) also exhibited fewer pawing events than B6 NS (*p* = 0.04), indicating that chronic tear deficiency, surgical manipulation, aging, and acute DS all dampen capsaicin-evoked pawing.

In contrast, Mmp9KO DS5 (15.10 ± 6.28 pawing/min) display numerically higher pawing than B6 NS, although this difference is not statistically significant (*p* = 0.13). Together, these findings suggest that while TRPV1 activation reliably elicits pawing in wild-type controls, nocifensive forepaw behavior is variably suppressed in chronic or surgically manipulated dry eye models, with a tendency toward enhanced pawing in the Mmp9KO under DS.

#### 3.3.2 Hypertonic saline

**Figure 5:**
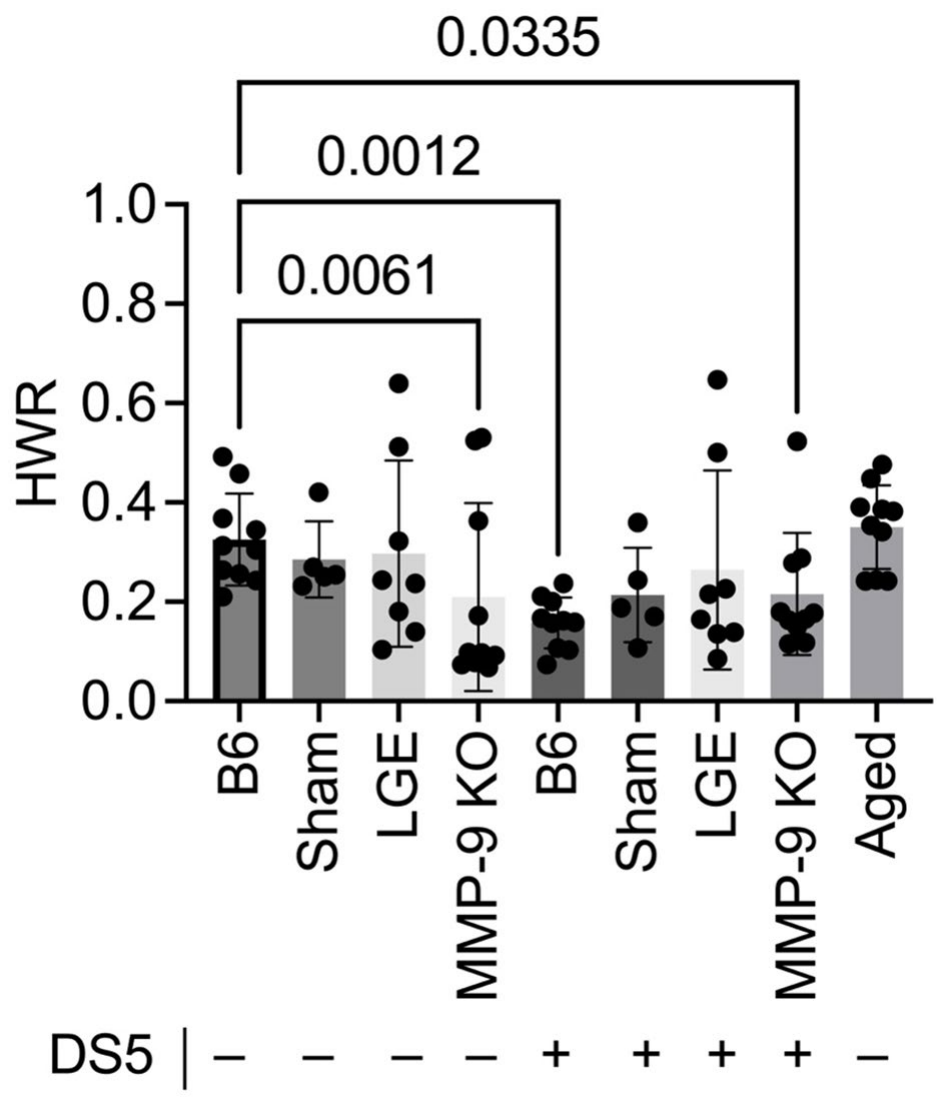
Ocular responses to hypertonic saline relative to hypertonic saline–treated B6 NS. Palpebral aperture following topical hypertonic saline challenge was measured in B6 and Mmp9KO mice under non stressed (NS) and desiccating stress for 5 days (DS5) conditions, as well as in sham-operated, lacrimal gland excision, and aged groups, as indicated. Data are shown as mean ± SD, with each dot representing one eye. All groups were compared following hypertonic saline application to the B6 NS reference group. *p* values < 0.05 were considered statistically significant. *p-values disp*layed above brackets indicate pairwise comparisons between the groups connected by each bracket. Statistical analysis was performed using the Kruskal–Wallis test followed by Dunn’s post hoc test without correction for multiple comparisons.

HS produces model-dependent reductions in palpebral aperture (Figure 5) when compared with B6 NS + HS controls (0.33 ± 0.10). Acute DS elicits the strongest response among wild-type groups. B6 DS5 (0.16 ± 0.05, *p* = 0.001) show a significantly greater reduction in aperture relative to B6 NS + HS, indicating heightened osmotic responsiveness following short-term DS.

Chronic aqueous deficiency produces a milder phenotype. LGE (0.30 ± 0.19, *p* = 0.30) did not differ from B6 NS + HS, and the addition of LGE DS_LH_ (0.26 ± 0.20, *p* = 0.07) likewise fails to generate further narrowing. Sham-operated animals behaved similarly. Sham Sx (0.28 ± 0.08, *p* = 0.72) and Sham Sx DS_LH_ (0.21 ± 0.09, *p* = 0.11) show no significant difference from B6 NS + HS, suggesting that surgical manipulation alone does not strongly modify osmotic sensitivity.

The response in aged mice (0.35 ± 0.08, *p* = 0.76) is comparable to B6 NS + HS.

Genotype exerted a distinct effect. Mmp9KO (0.21 ± 0.19, *p* = 0.006) exhibit significantly reduced apertures compared to B6 NS + HS, indicating increased baseline sensitivity to a hyperosmotic stimulus. Mmp9KO DS5 (0.22 ± 0.12, *p* = 0.03) show a similar reduction relative to B6 NS + HS, but difference between KO NS to KO DS5 was small, suggesting that the absence of *Mmp9* elevates osmotic responsiveness at baseline and limits further modulation by DS.

Blink behavior remained relatively stable across most models, with no significant differences between B6 NS + HS and B6 DS5, LGE, LGE DS_LH_, aged, Mmp9KO, Mmp9KO DS5 (all *p* > 0.3). A notable exception was Sham Sx DS_LH_, which exhibited a significantly elevated blink response to osmotic challenge (*p* = 0.046).

Pawing responses were absent in all groups except Mmp9KO NS (0.70 ± 1.57 pawing/min), which demonstrated significantly greater pawing activity than B6 NS + HS (0.00 pawing/min, *p* = 0.0006). No additional group showed significant HS-evoked nocifensive pawing (all *p* > 0.99). These findings indicate that osmotic nociception is selectively amplified in *Mmp9*–deficient animals, whereas the other dry eye models exhibit minimal forepaw-mediated nocifensive responses to HS.

#### 3.3.3 Menthol

**Figure 6:**
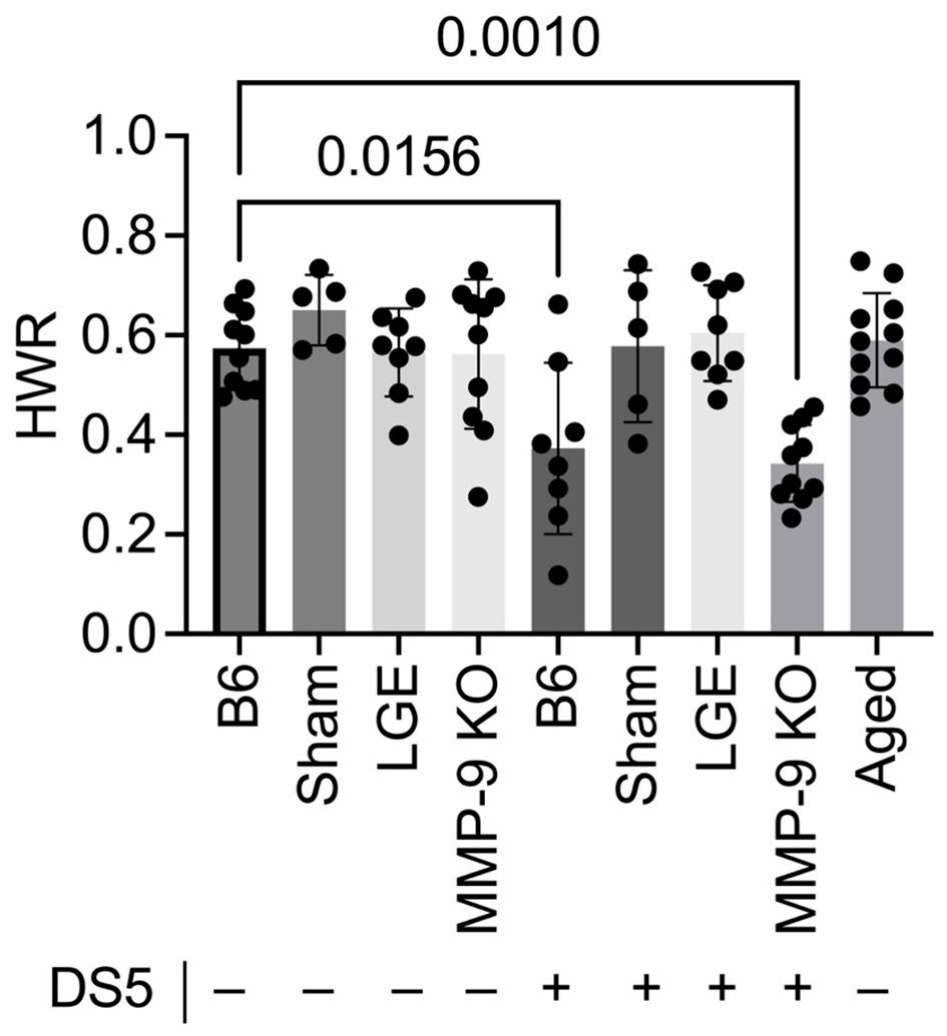
TRPM8-mediated sensory responses relative to menthol-treated B6 NS. Palpebral aperture following topical menthol application was measured in wild-type B6 and Mmp9KO mice under non stressed (NS) and desiccating stress for 5 days (DS5) conditions, as well as in sham-operated, lacrimal gland excision, and aged groups, as indicated. Data are shown as mean ± SD, with each dot representing one eye. All groups were compared following menthol application to the B6 NS reference group. *p* values < 0.05 were considered statistically significant. *p-values disp*layed above brackets indicate pairwise comparisons between the groups connected by each bracket. Statistical analysis was performed using the Kruskal–Wallis test followed by Dunn’s post hoc test without correction for multiple comparisons.

Application of menthol, a TRPM8 agonist, produces model-dependent variations in eyelid aperture (Figure 6), with B6 NS + menthol (0.57 ± 0.08) serving as the reference group. B6 DS5 (0.37 ± 0.17) exhibits a significant reduction in aperture relative to controls (*p* = 0.016), indicating enhanced TRPM8-driven eyelid narrowing after acute DS. A similar but more pronounced reduction occurs in Mmp9KO DS5 (0.34 ± 0.08, *p* = 0.001), revealing increased menthol sensitivity under stress in the absence of MMP-9. By contrast, menthol responses in Mmp9KO NS, Sham Sx NS, LGE NS, Sham Sx DS_LH_, LGE DS_LH_, and aged mice do not differ significantly from B6 NS + menthol (all *p* > 0.23), demonstrating that TRPM8 responsiveness is selectively heightened only in acute stress and *Mmp9* deficiency, but remains stable in chronic tear deficiency, surgical controls, and aging.

Blink responses following menthol application revealed a TRPM8-dependent suppression in specific groups. Compared with B6 NS controls (4.10 ± 3.21 blink/min), Mmp9KO NS (0.40 ± 0.97 blink/min, *p* = 0.002), B6 DS5 (0.00 blink/min, *p* = 0.0006), and Mmp9KO DS5 (0.00 blink/min, *p* = 0.0003) all exhibited significantly reduced blink rates. In contrast, Sham Sx, LGE, Sham Sx DS5, LGE DS5, and aged mice showed blink frequencies comparable to B6, indicating that menthol-evoked hypoblinking is selectively associated with desiccating stress and Mmp9 deficiency.

Pawing behavior is absent across all menthol-treated groups, indicating that TRPM8 activation does not elicit nocifensive forepaw responses under any condition and supporting the interpretation that menthol’s effects were limited to eyelid- and blink-related pathways.

The relative change in HWR produced by the three chemical stimulants in dry eye is shown in Supplementary Figure S3. Capsaicin produced the greatest reduction and menthol the least.

### 3.4 Model-specific sensory response profiles

In B6 DS5, chemical stimulation produced modality-specific responses when compared with B6 NS baseline controls. Capsaicin elicits a profound reduction in palpebral aperture (0.07 ± 0.03 vs. 0.77 ± 0.05, *p* < 0.0001), indicating strong TRPV1-mediated eyelid narrowing under acute DS. Hypertonic saline similarly causes a significant decrease in aperture (0.16 ± 0.05, *p* = 0.0001), consistent with heightened osmotic sensitivity in DS5 mice. Menthol produces a weaker but still significant effect (0.37 ± 0.17, *p* = 0.0180), reflecting a modest TRPM8-dependent response relative to TRPV1 and osmotic activation.

Blink responses show a distinct pattern. Capsaicin (3.12 ± 5.71 blink/min) does not significantly increase blinking relative to B6 NS baseline (0.00 blink/min, *p* = 0.0734), whereas HS induces a clear blink response (3.20 ± 5.29 blink/min, *p* = 0.012). Menthol fails to alter blink frequency (0.00 blink/min, *p* = > 0.9999), demonstrating that DS5 selectively activates blink pathways in response to one (HS) TRPV1 agonist, but not to the TRPM8 agonist.

Forepaw pain-related responses were minimal. Pawing remained absent in B6 NS baseline, but capsaicin triggered a small yet significant pawing increase in B6 DS5 (0.38 ± 0.52 pawing/min, *p* = 0.003). Neither HS nor menthol elicited pawing (both 0.00 pawing/min, *p* = > 0.9999).

Chemical stimulation elicited robust eyelid and blink responses in chronic tear-deficient and sham-operated mice when compared with B6 NS baseline. Following capsaicin application, LGE has a pronounced reduction in palpebral aperture (0.40 ± 0.09, *p* = 0.0003), while Sham Sx also exhibited significant aperture narrowing (0.45 ± 0.11, *p* = 0.013). When DS was superimposed, LGE DS_LH_ demonstrated an even stronger capsaicin-evoked effect (0.46 ± 0.17, *p* = 0.005), whereas Sham Sx DS_LH_ (0.53 ± 0.19, *p* = 0.19) showed no detectable change, suggesting that LGE sensitizes the TRPV1 response in a manner that is further amplified by DS.

Hypertonic saline produced a similar pattern. LGE + HS responses (0.30 ± 0.19, *p* = 0.0006) and LGE DS_LH_ + HS responses (0.26 ± 0.20, *p* < 0.0001) were significantly reduced compared with B6 NS baseline, indicating enhanced osmotic sensitivity in the context of LGE and DS. Sham Sx showed a smaller but still significant reduction (0.28 ± 0.08, *p* = 0.02). Sham Sx DS_LH_ also demonstrated a strong HS-evoked response (0.21 ± 0.09, *p* = 0.0006). These findings suggest that both surgery and desiccating environmental stress independently potentiate responses to hyperosmolar stimulation.

Menthol stimulation induces consistent reductions in aperture across LGE models. LGE menthol responses (0.57 ± 0.09, *p* = 0.0006) are significantly lower than baseline, and LGE DS_LH_ (0.60 ± 0.10, *p* = 0.005) shows similar sensitivity, indicating that TRPM8 responsiveness is preserved in chronic tear deficiency. Sham Sx mice showed no significant menthol effect (0.65 ± 0.07,*p* = 0.13), whereas Sham Sx DS_LH_ mice exhibited a modest but significant response (0.58 ± 0.15, *p* = 0.019).

Blink dynamics complemented aperture changes. Capsaicin produced large and highly significant blink increases in LGE (8.75 ± 4.27 blink/min, *p* = 0.0011), Sham Sx (11.0 ± 4.90 blink/min, *p* = 0.0007), and particularly LGE DS_LH_ mice (13.38 ± 5.90 blink/min, *p* < 0.0001), indicating strong TRPV1-driven activation. A similar pattern was observed following HS. Significant blink elevations occurred in LGE (3.50 ± 3.50 blink/min), Sham Sx (3.40 ± 2.30 blink/min), LGE DS_LH_ (3.62 ± 2.72 blink/min), and Sham Sx DS_LH_ (8.00 ± 7.18 blink/min, all *p* < 0.03). Menthol evoked significant blink responses across all groups, including LGE (4.75 ± 2.61 blink/min, *p* = 0.0002), Sham Sx (6.40 ± 6.73 blink/min, *p* = 0.001), LGE DS_LH_ (4.50 ± 4.34 blink/min, *p* = 0.002), and Sham Sx DS_LH_ (4.60 ± 6.10 blink/min, *p* = 0.02). These results indicate that both surgical manipulation and DS potentiate TRPM8-driven blinking, though the magnitude of the response varied across models.

Pawing responses are absent under all conditions (all means = 0), demonstrating that while capsaicin, HS, and menthol elicit strong eyelid and blink responses in LGE- and sham-derived models, they do not trigger pawing behavior in these groups.

In aged B6 mice, chemical stimulation produces selective alterations in eyelid aperture and blink activity relative to aged baseline controls. Capsaicin evokes a marked reduction in palpebral aperture (0.45 ± 0.13 vs. 0.73 ± 0.11, *p* = 0.0009), indicating that TRPV1 sensitivity remains robust in older animals despite age-related epithelial changes. Hypertonic saline induces an even stronger effect (0.35 ± 0.08, *p* < 0.0001), demonstrating preserved and heightened osmotic responsiveness in aged corneas. In contrast, menthol does not significantly alter aperture (0.59 ± 0.09, *p* = 0.09), suggesting that TRPM8-mediated cooling pathways are relatively stable with aging.

Blink responses followed similar modality-specific pattern. Capsaicin produces a large and significant increase in blink frequency (4.70 ± 2.75 vs. 0 blink/min, *p* < 0.0001), whereas HS elicits only a mild, non-significant effect (1.50 ± 1.18 blink/min, *p* = 0.055). Menthol induces a significant increase in blinks (3.42 ± 3.50 blink/min, *p* = 0.0014), indicating that aged mice maintain robust blink reactivity to capsaicin and TRPM8 stimulation but exhibit weaker activation in response to osmotic challenge.

Pawing responses remained absent under all conditions (all means = 0), confirming that chemical stimulation did not elicit pawing behavior in aged mice.

In the Mmp9KO strain under non-stressed conditions, chemical stimulation elicits strong modality-specific responses when compared with Mmp9KO baseline controls. Capsaicin produces a striking reduction in palpebral aperture (0.21 ± 0.19 vs. 0.70 ± 0.08, *p* < 0.0001), indicating that TRPV1-driven eyelid narrowing is markedly amplified in the absence of MMP-9 even without DS. HS induces a similarly strong aperture decrease (0.20 ± 0.08, *p* < 0.0001), demonstrating significant osmotic hypersensitivity in MMP-9 deficient corneas. In contrast, menthol does not significantly alter aperture (0.56 ± 0.15, *p* = 0.16), suggesting that TRPM8-mediated cooling pathways remain largely intact in KO strain.

Blink activity further highlighted the selective enhancement of TRPV1 signaling in this genotype. Capsaicin triggers a large increase in blink frequency (11.20 ± 9.94 blink/min, *p* = 0.0001), whereas neither HS (1.50 ± 1.84 blink/min, *p* = 0.18) nor menthol (0.40 ± 0.97 blink/min, *p* = 0.92) produced significant changes relative to KO baseline. These findings indicate strong TRPV1, but not osmotic or TRPM8-driven blink activation in KO animals.

Pawing responses show an even more pronounced divergence. Capsaicin evokes a substantial increase in pawing (10.80 ± 11.72 vs. 0.00 pawing/min, *p* = 0.0008), whereas HS induces only a minimal and non-significant increase (0.70 ± 1.57 pawing/min, *p* = 0.17) and menthol fails to elicit pawing (0.00 pawing/min, *p* > 0.9999). This pattern demonstrates that TRPV1 activation with capsaicin uniquely triggers nocifensive forepaw activity in *Mmp9* deficient mice, whereas osmotic and TRPM8 pathways do not induce a pawing response under non-stressed conditions.

Chemical stimulation produces pronounced modality-specific effects relative to KO NS baseline controls (0.70 ± 0.08) in Mmp9KO exposed to DS. Capsaicin evokes a dramatic reduction in palpebral aperture (0.07 ± 0.01, *p* < 0.0001), indicating marked TRPV1 hypersensitivity in the KO background following DS5. HS also produces a significant aperture reduction (0.22 ± 0.12, *p* = 0.0004), reflecting potent osmotic responsiveness under stress. Menthol induced a modest but significant decrease in aperture (0.34 ± 0.08, *p* = 0.03), suggesting that TRPM8 sensitivity, while preserved, is less strongly engaged than TRPV1 pathways in Mmp9KO DS5 mice.

Blink responses follow a parallel pattern. Capsaicin triggers a large blink increase relative to KO NS baseline (6.50 ± 2.99 blink/min, *p* < 0.0001), and HS elicits a smaller but significant elevation (2.20 ± 2.35 blink/min, *p* = 0.03). In contrast, menthol produced no change in blinking (0.00 blink/min, *p* > 0.9999), indicating that blink activation in Mmp9KO DS5 mice is selectively driven by TRPV1 and osmotic pathways, but not by TRPM8 stimulation.

Pawing activity shows a striking TRPV1 specificity. Pawing remained absent under baseline conditions, yet capsaicin induces a robust increase in pawing behavior (15.10 ± 6.28 pawing/min, *p* < 0.0001). Neither HS nor menthol produced detectable pawing (both 0.00, *p* > 0.9999), demonstrating that TRPV1, but not TRPM8 stimuli, is the primary driver of nocifensive pawing responses in Mmp9KO DS5.

### 3.5 Neurosensory impact of corneal epithelial debridement

**Figure 7:**
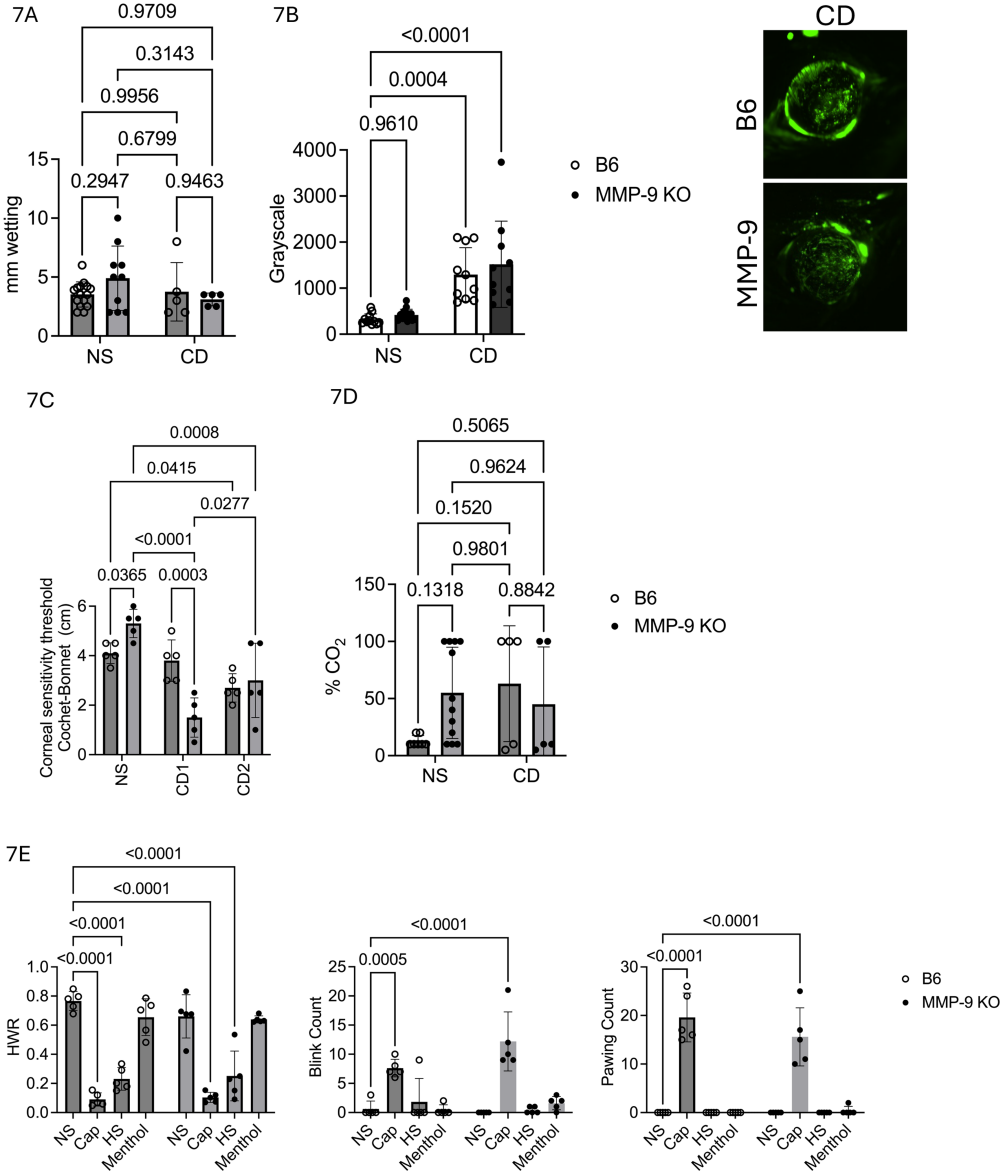
Neurosensory impact of corneal epithelial debridement. Corneal neurosensory function was evaluated in wild-type B6 and Mmp9KO mice under non stressed (NS) conditions and following corneal epithelial debridement (CD). Data are shown as mean ± SD, with each dot representing one eye. **(A)** Tear secretion measured by phenol red thread in B6 and Mmp9KO mice under NS and CD conditions. **(B)** Corneal epithelial permeability assessed by OGD fluorescence (grayscale intensity) under NS and CD conditions, with representative corneal surface fluorescence images shown at right. **(C)** Corneal mechanical sensitivity thresholds measured by Cochet–Bonnet esthesiometry in NS, 24 h after corneal epithelial debridement (CD1), and 48 h after corneal epithelial debridement (CD2) conditions. **(D)** CO₂ sensitivity under NS and CD conditions. **(E)** Behavioral and reflex responses including palpebral aperture, blink count, and pawing count following topical chemical stimuli (capsaicin, hypertonic saline, and menthol) under NS and CD conditions. *p* values < 0.05 were considered statistically significant. *p-*values displayed above brackets indicate pairwise comparisons between the groups connected by each bracket. Panels A, C, and D were analyzed using two-way ANOVA with Tukey’s multiple comparisons test, in which all groups were compared with each other. Panels B and E were analyzed using two-way ANOVA with Sidak’s multiple comparisons test, in which comparisons were made relative to the B6 NS reference group.

Corneal epithelial debridement does not significantly alter aqueous tear secretion (Figure 7A). Tear volume in debrided B6 mice (3.75 ± 2.49 mm) is similar to B6 NS baseline controls (3.53 ± 1.07 mm, *p* = 0.99). Similarly, debrided Mmp9KO (3.10 ± 0.55 mm) do not differ from their NS counterparts (4.90 ± 2.73 mm, *p* = 0.31) or from debrided B6 mice (*p* = 0.95). No interaction effects between genotype and injury were detected. These findings demonstrate that acute epithelial removal does not impair tear production, indicating that the sensory abnormalities observed after debridement are independent of changes in aqueous tear output.

Corneal debridement produces a time-dependent decline in mechanical sensitivity (Figure 7C) that differs markedly between wild-type and Mmp9KO mice. In B6 animals, sensitivity declined modestly at 24 h (3.8 ± 0.84 vs. 4.1 ± 0.42 cm, *p* = 0.85) and became significantly reduced by 48 h (2.7 ± 0.57 cm, *p* = 0.04). This indicates a progressive impairment of mechanoreceptor function as epithelial injury evolves from acute to subacute phases.

In contrast, Mmp9KO mice show an exaggerated early loss of sensitivity, with CD1 producing a large and highly significant reduction (1.5 ± 0.79 vs. 5.3 ± 0.57 cm, *p* < 0.0001). At 48 h, sensitivity partially recovers (3.0 ± 1.50 cm) but remains significantly lower than KO baseline (*p* = 0.0008) (Figure 7C).

Direct comparisons between genotypes further highlighted divergent trajectories. At baseline, Mmp9KO NS show slightly higher sensitivity than B6 NS (*p* = 0.04). At 24 h CD1, KO are dramatically less sensitive than B6 (*p* = 0.0003). By 48 h CD2, genotype differences disappear (*p* = 0.58), reflecting partial recovery in KO mice and continued decline in WT.

Together, these findings demonstrate that corneal debridement produces a biphasic mechanical desensitization that is stronger and earlier in *Mmp9*–deficient mice but converges across genotypes by 48 h post-injury.

Unlike mechanical sensitivity, CO₂-evoked chemosensory responses does not show significant alterations following corneal debridement (Figure 7D). In B6, CO₂ thresholds increase from baseline (12.86 ± 4.88 mL/min) to debridement (63.0 ± 50.70 mL/min), but this difference is not statistically significant (*p* = 0.15). Similarly, Mmp9KO display elevated but highly variable thresholds after debridement (45.0 ± 50.25 vs. 55.0 ± 39.89 mL/min, *p* = 0.96).

Between-group comparisons confirmed that neither genotype exhibits significant hypersensitivity or hyposensitivity following epithelial removal (all *p* > 0.13).

Thus, while mechanical sensitivity was strongly affected by epithelial debridement, CO₂ chemosensory responsiveness remained largely unchanged, suggesting that polymodal nociceptors are less perturbed by epithelial injury than mechanoreceptors.

Corneal debridement causes a profound loss of epithelial barrier integrity (Figure 7B), reflected by a dramatic increase in OGD fluorescence intensity compared with non-injured corneas. In B6, OGD levels rise from 324.5 ± 118.6 at baseline to 1295.5 ± 583.1 gray levels after debridement, representing a highly significant elevation (*p* = 0.0008). A similar large increase occurs in Mmp9KO, rising from 418.1 ± 124.0 at baseline to 1518.7 ± 937.6 after injury (*p* < 0.0001). Genotype does not significantly modify the magnitude of barrier disruption, as OGD levels after debridement do not differ between B6 and Mmp9KO strains (*p* = 0.92).

Consistent with these findings, both B6 CD and KO CD groups exhibit substantially higher OGD values than their respective non-stressed controls (all *p* ≤ 0.0014), demonstrating that epithelial debridement induces the greatest barrier disruption across all experimental models. Baseline OGD values do not differ between genotypes (*p* = 0.99), confirming that the observed increase is injury-specific rather than genotype-driven.

Together, these results show that corneal epithelial debridement produces severe, genotype-independent epithelial barrier disruption, far exceeding the permeability changes seen in the DS or chronic aqueous-deficient models.

Corneal debridement produced strong alterations in spontaneous eyelid dynamics (Figure 7E), with modality-specific effects on palpebral aperture, blink activity, and pawing behavior. Compared with B6 NS baseline (0.77 ± 0.07), palpebral aperture was profoundly reduced in both genotypes following capsaicin stimulation (B6: 0.09 ± 0.04, KO: 0.10 ± 0.03, both *p* < 0.0001), indicating a strong TRPV1-mediated eyelid-closure response after injury. HS also significantly narrowed the aperture in B6 (0.23 ± 0.08) and KO (0.25 ± 0.17, both *p* < 0.0001). In contrast, menthol failed to alter HWR relative to baseline in either genotype (B6: *p* = 0.66, KO: *p* = 0.50), suggesting preserved TRPM8-mediated tonic aperture control.

Blinking responses exhibit a distinct pattern. Capsaicin evokes a marked increase in blink frequency in both B6 (7.6 ± 1.52 blink/min, *p* = 0.0005) and Mmp9KO (12.2 ± 5.07 blink/min, *p* < 0.0001), confirming heightened TRPV1-dependent excitability after debridement (Figure 7E). However, neither HS nor menthol produce significant blink changes relative to baseline (all *p* > 0.95), indicating selective activation of blink circuitry by TRPV1, but not osmotic or cold pathways.

Pawing responses are strongly enhanced exclusively by capsaicin (Figure 7E). Pawing remained absent under baseline conditions (0.00 pawing/min in both genotypes), but increased sharply after TRPV1 activation in B6 (19.6 ± 5.03 pawing/min, *p* < 0.0001) and KO (15.6 ± 5.98 pawing/min, *p* < 0.0001). Neither HS nor menthol evoked pawing (all *p* > 0.99), indicating that only TRPV1 stimulation engages these nociceptive pathways after debridement, while osmotic and TRPM8 stimuli are insufficient to trigger forelimb defensive behavior.

Together, these findings show that corneal debridement dramatically heightens TRPV1-driven eyelid closure, blink activity, and nocifensive responses, while leaving response to TRPM8 agonist and osmotic stress functionally intact or only modestly affected.

## 4 Discussion

DED is increasingly recognized as a disorder of ocular sensory homeostasis in which tear film instability, epithelial barrier compromise, and neural dysregulation converge to drive symptoms. Prior clinical and experimental studies have demonstrated that distinct dry eye etiologies produce divergent sensory signatures rather than a single unified pain pathway (Bron et al., 2017; Saram et al., 2025). The present work integrates complementary human functional testing (including eye aspect ratio and blink frequency under baseline and evaporative stress conditions) with mouse behavioral assays across DE, lacrimal gland deficiency, and epithelial injury, revealing discrete patterns of TRPV1-, TRPM8-, and mechanoreceptor-driven responses. These findings provide mechanistic insight into the heterogeneous sensory phenotypes observed in patients and highlight the value of stimulus-specific readouts for distinguishing underlying disease mechanisms.

Our human data confirm that patients with DED exhibit reduced palpebral aperture and heightened blink frequency even without environmental stress. These observations are consistent with prior clinical imaging and TFOS DEWS II analyses showing exaggerated environmental sensitivity and increased blink instability in symptomatic patients despite modest clinical surface findings (Su et al., 2018). TRPM8-expressing cold thermoreceptors, which monitor tear film thinning and initiate compensatory blinking and tear production, are known to fire more readily in unstable tear environments (Quallo et al., 2015). The reduction in EAR and increased blink rate in our cohort may reflect altered ocular surface sensory processing, including increased responsiveness of TRPM8-mediated pathways involved in tear film regulation and blinking (Belmonte and Gallar, 2011; Quallo et al., 2015). Consistent with these findings, baseline palpebral aperture was also reduced in DS5 and LGE mouse models, indicating that similar alterations in ocular surface function are observed across species. Importantly, while direct equivalence between mouse models and human disease is not expected, the alignment of stimulus-specific functional outputs supports the translational relevance of these models and provides a clinically meaningful context for interpreting sensory changes across systems.

In mice, acute DS5 dry eye model produced the strongest capsaicin-evoked narrowing of the palpebral aperture, together with markedly elevated hypertonic saline responses, supporting increased TRPV1-mediated sensitivity under inflammatory conditions (Masuoka et al., 2020). In addition to capsaicin and hypertonic saline, menthol also reduced palpebral aperture under desiccating stress conditions, consistent with a broad enhancement of stimulus-evoked eyelid responses. These findings mirror longstanding evidence that DS enhances TRPV1 expression, promotes inflammatory cytokine release, and sensitizes corneal polymodal nociceptors (Li et al., 2019; Pan et al., 2011). Similar enhancements in polymodal nociceptor activity have been reported in inflammatory models such as photokeratitis (Acosta et al., 2014) and allergic keratoconjunctivitis (Acosta et al., 2013), supporting the role of inflammation-driven TRPV1 sensitization in corneal pain. Human patients experiencing “burning pain” in DED often exhibit elevated tear osmolarity and MMP-9 levels, and respond favorably to anti-inflammatory therapy. These findings mechanistically align with TRPV1-mediated hyperexcitability (Gou et al., 2024; Pflugfelder et al., 2017). The DS5 phenotype in our study has similar features to inflammatory nociceptive sensitivity described clinically, supporting its validity as a translational model for symptomatic DED. Consistent with this inflammatory milieu, desiccating stress has also been shown to reduce corneal intraepithelial nerve density (Stepp et al., 2018), providing a structural correlate for the significance sensory dysfunction observed in DS-exposed animals (Table 1). These findings are consistent with prior studies of inflammatory dry eye models and further support the concept that TRPV1-driven sensitization is a key mechanism underlying burning discomfort or pain experienced by patients with DED (Belmonte et al., 2004; Belmonte et al., 2017). Notably, hypertonic saline–evoked responses in our study did not fully mirror capsaicin responses, but instead exhibited an intermediate profile, consistent with engagement of multiple sensory pathways rather than a purely TRPV1-mediated effect. This is further supported by evidence that corneal afferents may co-express TRPV1 and TRPM8, enabling overlapping or mixed sensory outputs when exposed to hypertonic conditions (Bereiter et al., 2018; Li et al., 2019). This intermediate profile was also reflected in the magnitude of palpebral aperture reduction observed in the acute DS5 dry eye model, which falls between capsaicin- and menthol-evoked responses.

LGE induced dry eye displayed reduced TRPV1-mediated responsiveness, together with preserved or exaggerated blink responses to hyperosmolar stress and menthol, respectively, indicating a possible shift toward TRPM8-associated sensory processing. Importantly, the reduced responses observed in sham-operated animals suggest that surgical manipulation alone may modulate corneal sensory function, supporting the interpretation that the LGE phenotype reflects both procedural and gland-specific effects. Notably, hypertonic saline responses were not significantly altered in LGE, further supporting a dissociation between TRPV1-mediated and osmolarity-driven responses in chronic dry eye (Fakih et al., 2022). These findings are in contrast to what was found in the rat lacrımal gland excısıon model whıch showed hypersensıtıvıty to capsaicin (Bereiter et al., 2018). This difference may be related to the capsaicin concentration used in our studies that was titrated to reduce palpebral aperture, but not induce prolonged eye closure in non-stressed animals. Studies of aqueous deficient dry eye have reported decreased mechanical, chemical, and thermal sensitivity despite increased patient reported symptoms of eye dryness and irritation (Belmonte et al., 2017; Bourcier et al., 2005). Response to the chemical stimulants used in this study has not been systematically evaluated in human patients, but this might prove valuable in assessing the neurosensory impact of dry eye. Importantly, prior morphologic and our studies (Supplementary Figure S4) have found no significant differences in corneal subbasal nerve density between LGE and sham-operated animals, indicating that the altered sensory phenotype in LGE is driven primarily by altered sensory thresholds rather than structural nerve loss (Mecum et al., 2020). Taken together, these findings highlight a clear divergence between acute desiccating stress and chronic lacrimal gland deficiency, with acute models showing enhanced TRPV1 and osmotic responsiveness, whereas chronic tear deficiency is associated with attenuated TRPV1 signaling and preserved or selectively altered TRPM8-mediated pathways.

Previous work from our group has demonstrated that corneal barrier integrity declines with age. McClellan et al. (2014) reported that middle-aged female mice exhibited significantly increased permeability to the fluorescent 70-kDa tracer OGD compared with young females and age-matched males, consistent with progressive age-related epithelial fragility. Although OGD staining was not repeated in aged mice in the present study, these published findings provide important context for interpreting age-associated ocular surface changes, particularly given that aged B6 mice in our cohort exhibited increased tear volume. In parallel, several studies have documented age-associated reductions in corneal subbasal nerve density and nerve fiber complexity, indicating that sensory alterations in aged mice reflect intrinsic epithelial and neural remodeling rather than aqueous tear deficiency, which is preserved in aging (Stepp et al., 2018). Together, these findings demonstrate that corneal sensory dysfunction across dry eye, epithelial injury, and aging emerges from distinct combinations of tear film instability, epithelial barrier compromise, and neural remodeling, rather than from a single uniform “dryness” mechanism. This variability is consistent with prior reports showing that aging-related sensory changes are not uniformly directional (Bron et al., 2017; Vereertbrugghen and Galletti, 2022).

Genetic deletion of Mmp9 resulted in a distinct neurosensory phenotype. While capsaicin-evoked eyelid narrowing did not differ significantly between Mmp9KO and wild-type mice, Mmp9KO animals exhibited reduced blink responses and variable pawing behavior following TRPV1 activation. This dissociation suggests that Mmp9 deficiency alters the coupling between corneal nociceptor activation and downstream motor responses, revealing distinct regulatory layers in TRPV1-mediated sensory processing. Previous molecular studies have shown that MMP-9 facilitates epithelial barrier disruption, cytokine penetration, and exposure of corneal nerve endings, key steps in nociceptor sensitization under DS (De Paiva et al., 2009; Pflugfelder et al., 2005). Clinically, tear MMP-9 is elevated in painful DED and correlates with symptom severity and hyperalgesia (Lanza et al., 2016; Messmer et al., 2016). Our findings provide functional support for this clinical dichotomy, suggesting that MMP-9 is not merely a biomarker but an active modulator of polymodal nociception.

Mechanical sensitivity was reduced across dry eye models, reflecting diminished mechanoreceptive Aδ fiber function associated with ocular surface stress and epithelial disruption (Belmonte et al., 2004; Bourcier et al., 2005; Craig et al., 2017). Chemical sensitivity, however, showed a modality-specific pattern. Notably, CO₂-evoked responses should be interpreted within the context of tear film dynamics, as this stimulus depends on tear film–mediated acidification and reflects an integrated sensory response rather than a purely chemical input (Belmonte et al., 2004; Merino et al., 2023; Yang et al., 2018). This composite nature may also explain why CO₂-evoked responses do not directly parallel those observed with capsaicin, as the CO₂ stimulus integrates chemical, evaporative, and thermal components. We observed that CO₂-evoked sensitivity remained largely unchanged across dry eye models, except in Mmp9KO mice where a reduction was observed. This altered response in Mmp9KO may relate to the reduced epithelial barrier disruption observed in this strain, suggesting that preservation of barrier integrity can influence stimulus-evoked sensory signaling. In contrast to its protective effects in desiccating stress–induced barrier disruption, Mmp9 deletion did not significantly alter sensory responses following epithelial debridement in our study.

Consistent with this modality-specific dissociation, corneal epithelial debridement further highlighted divergence between mechanical and chemical pathways. Capsaicin-evoked responses were markedly enhanced after debridement, whereas CO₂-evoked polymodal nociceptor activity did not increase, indicating that epithelial injury selectively facilitates TRPV1-mediated hyperalgesia rather than broad chemical hypersensitivity.

Comparable dissociations between reduced mechanical sensitivity and increased chemical hyperalgesia have been described in injury models such as microkeratome-induced epithelial disruption, further supporting modality-specific sensory remodeling following corneal injury (Belmonte et al., 2004; Benitez-del-Castillo et al., 2001; Calvillo et al., 2004). Clinically, similar dissociations are well recognized after photorefractive keratectomy (PRK) (Gallar et al., 2007), corneal crosslinking (Kontadakis et al., 2013; Wasilewski et al., 2013), and infectious keratitis (Hamrah et al., 2010), where patients often report pronounced burning pain despite reduced mechanical sensitivity on esthesiometry, an effect attributed to enhanced TRPV1 drive in the setting of epithelial barrier loss (Rosenthal and Borsook, 2012). In our model, fluorescent tracer staining confirmed significant surface disruption after debridement, supporting the interpretation that epithelial compromise increases access of lipophilic agonists such as capsaicin to subepithelial nerve fibers.

When considered together, these findings support a three-module organizational framework for ocular sensory processing. The first module involves polymodal nociceptor–driven modulation of palpebral aperture, which is particularly sensitive to epithelial injury, inflammation, and TRPV1 activation. The second module reflects TRPM8-mediated evaporation sensing and reflex blinking, tightly linked to tear instability and ocular surface cooling. The third module encompasses high-threshold nocifensive behaviors such as pawing, representing deeper polymodal C-fiber engagement. Different dry eye etiologies selectively perturb these sensory units: DS activates all three modules; chronic lacrimal gland deficiency preferentially alters TRPM8-associated evaporation and blink pathways; epithelial injury amplifies polymodal nociceptor output while transiently suppressing mechanoreceptor function; and MMP-9 deletion differentially alters nociceptor-driven aperture signaling and high-threshold defensive responses. Together, this modular framework provides a mechanistic explanation for the divergent sensory phenotypes observed across dry eye models and highlights how specific disease mechanisms differentially shape the neural coding of ocular discomfort. This framework is further supported by prior electrophysiological and clinical studies demonstrating functional specialization among corneal sensory subtypes and context-dependent variability in their activation (Belmonte et al., 2004; Belmonte et al., 2017; Rosenthal and Borsook, 2012).

These findings emphasize that symptom profiles in DED arise not from a uniform “dryness” mechanism but from selective disruption of distinct sensory pathways. A sensory-based clinical classification: differentiating evaporative discomfort, inflammatory burning pain, injury-induced hyperalgesia, and neuropathic sensitization may substantially improve diagnostic precision and therapeutic targeting. Patients whose symptoms are dominated by evaporative or TRPM8-related dysfunction may respond best to tear film stabilizing treatments, scleral lenses to minimize cooling stimuli and TRPM8 agonists to stimulate tearing realizing they may cause instillation discomfort. Those experiencing TRPV1-mediated burning pain may require anti-inflammatory strategies or emerging TRPV1 modulators. Individuals whose discomfort is driven by epithelial injury could benefit more from surface-restorative or neurotrophic therapies aimed at rebuilding epithelial integrity and normalizing nociceptor exposure. By linking specific mouse models to corresponding human sensory phenotypes, the present work provides a translational framework for selecting preclinical systems that best align with distinct clinical endotypes, thereby enhancing the relevance and precision of therapeutic development.

### 4.1 Limitations and future directions

Although strong across modalities, our analysis does not encompass chronic remodeling beyond acute injury windows, nor does it examine sex differences, systemic inflammatory states, or higher-order central sensitization. Future studies integrating *in vivo* confocal microscopy, molecular profiling, and electrophysiological recordings will help clarify how specific corneal sensory neuron subtypes, such as mechanoreceptive Aδ fibers, polymodal C-nociceptors, and cold-sensitive TRPM8 neurons, individually contribute to the distinct behavioral responses observed in our models. Nonetheless, the strong convergence between clinical and experimental findings supports a unified mechanistic framework for interpreting DED sensory phenotypes.

## 5 Conclusion

In summary, our findings indicate that dry eye does not disrupt corneal sensation in a single, uniform way. Instead, each type of stress on the ocular surface, such as desiccation, lacrimal gland dysfunction, epithelial injury, aging, or loss of MMP-9, leads to distinct changes in corneal sensory processing.

Corneal sensory responses also depend strongly on the type of stimulus. Desiccating stress increases TRPV1- and osmolarity-related responses, lacrimal gland excision is associated with changes in TRPM8-linked evaporative feedback, and epithelial debridement produces a combination of reduced mechanical sensitivity and increased chemical responsiveness. The reduced responses observed in Mmp9KO mice across several conditions further support the idea that inflammation plays an active role in shaping ocular sensory function.

These findings suggest that similar clinical presentations in dry eye disease may arise from different underlying neural mechanisms. For example, symptoms such as burning pain, mechanical discomfort, or sensitivity to airflow may reflect reduced or increased sensitivity of different sensory pathways rather than a single common process.

By comparing mouse behavior with human eyelid dynamics, this study provides a link between experimental findings and clinically observable features. The parallel changes we observed, including reduced palpebral aperture and increased blink responses, are consistent with what is commonly seen in patients experiencing light sensitivity or discomfort in visually demanding or dry environments.

Overall, these results support a more mechanism-based understanding of dry eye disease and suggest that future treatment strategies may benefit from targeting specific sensory pathways, rather than relying only on general anti-inflammatory or tear replacement approaches.

## Data Availability

The original contributions presented in the study are included in the article/Supplementary material, further inquiries can be directed to the corresponding author.

## Glossary

ATD: Aqueous-deficient dry eye
ATD-SS: Aqueous-deficient dry eye associated with Sjögren’s syndrome
B6: C57BL/6J mice
C-B: Cochet–Bonnet esthesiometer
CD: Corneal debridement
CCh: Conjunctivochalasis
DED: Dry eye disease
DS: Desiccating stress
DS_LH_: DS with low humidity alone
DS5: 5-day desiccating stress
EAR: Eye aspect ratio (palpebral aperture ratio)
FTBUT: Fluorescein tear break-up time
HWR: Height-to-width ratio
HS: Hypertonic saline
IRB: Institutional Review Board
KO: Knockout
LGE: Lacrimal gland excision
MGD: Meibomian gland dysfunction
MMP-9: Matrix metalloproteinase-9
NK: Neurotrophic keratitis
NS: Non stressed
OGD: Oregon Green Dextran
PRK: Photorefractive keratectomy
RH: Relative humidity
ROI: Region of interest
SANDE: Symptom Assessment in Dry Eye questionnaire
Sham Sx: Sham surgery
TG: Trigeminal ganglion
TRP: Transient receptor potential
TRPM8: Transient receptor potential melastatin 8
TRPV1: Transient receptor potential vanilloid 1
WT: Wild-type
