# Supplementary figures and images for: Modulation of corneal sensory processing and pain responses by dry eye and corneal wounding
Source: Front Cell Neurosci. 2026 Jun 2;20:1767673. doi: 10.3389/fncel.2026.1767673 (PMC13268892; doi:10.3389/fncel.2026.1767673)

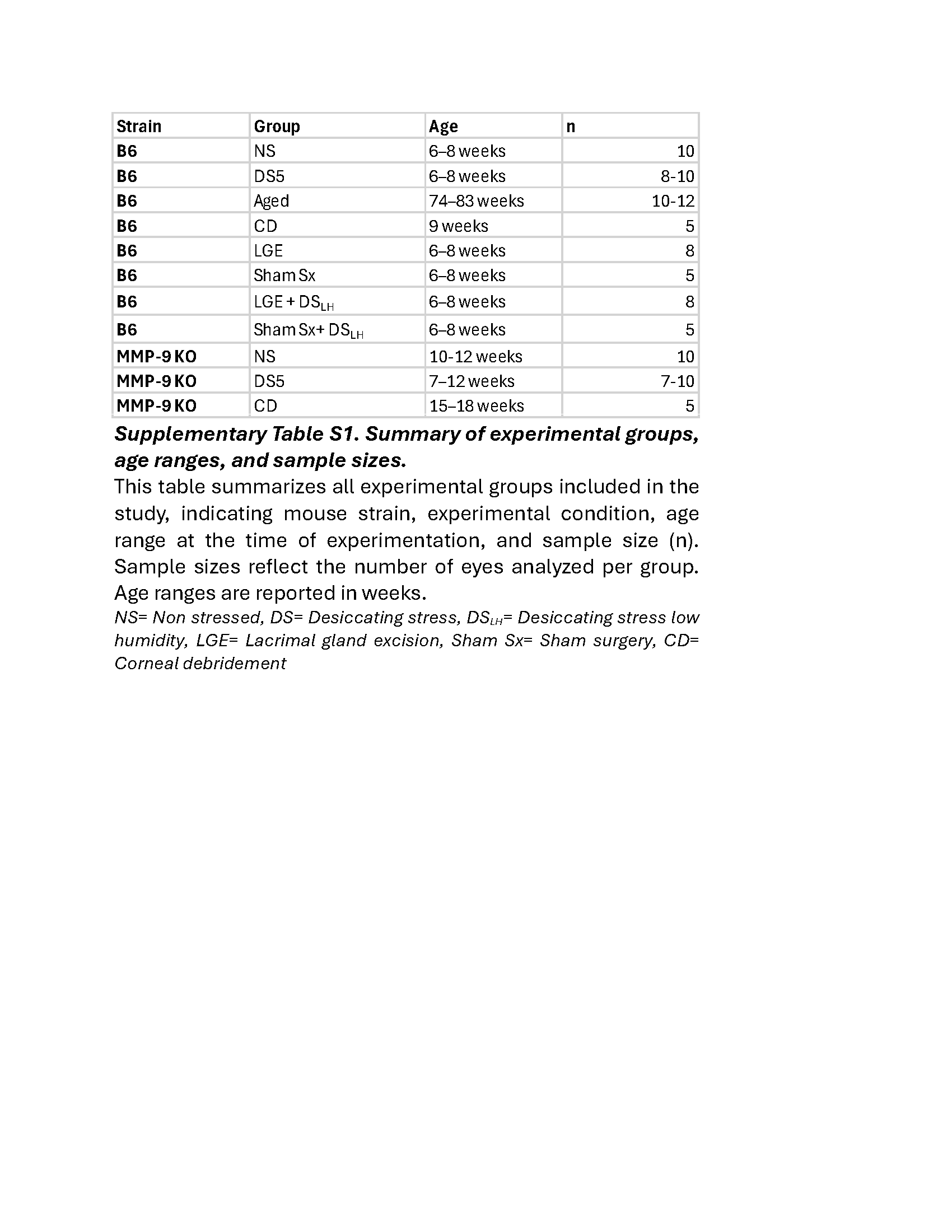

Supplement: Supplementary file 1 [file Supplementary_file_1.tiff]

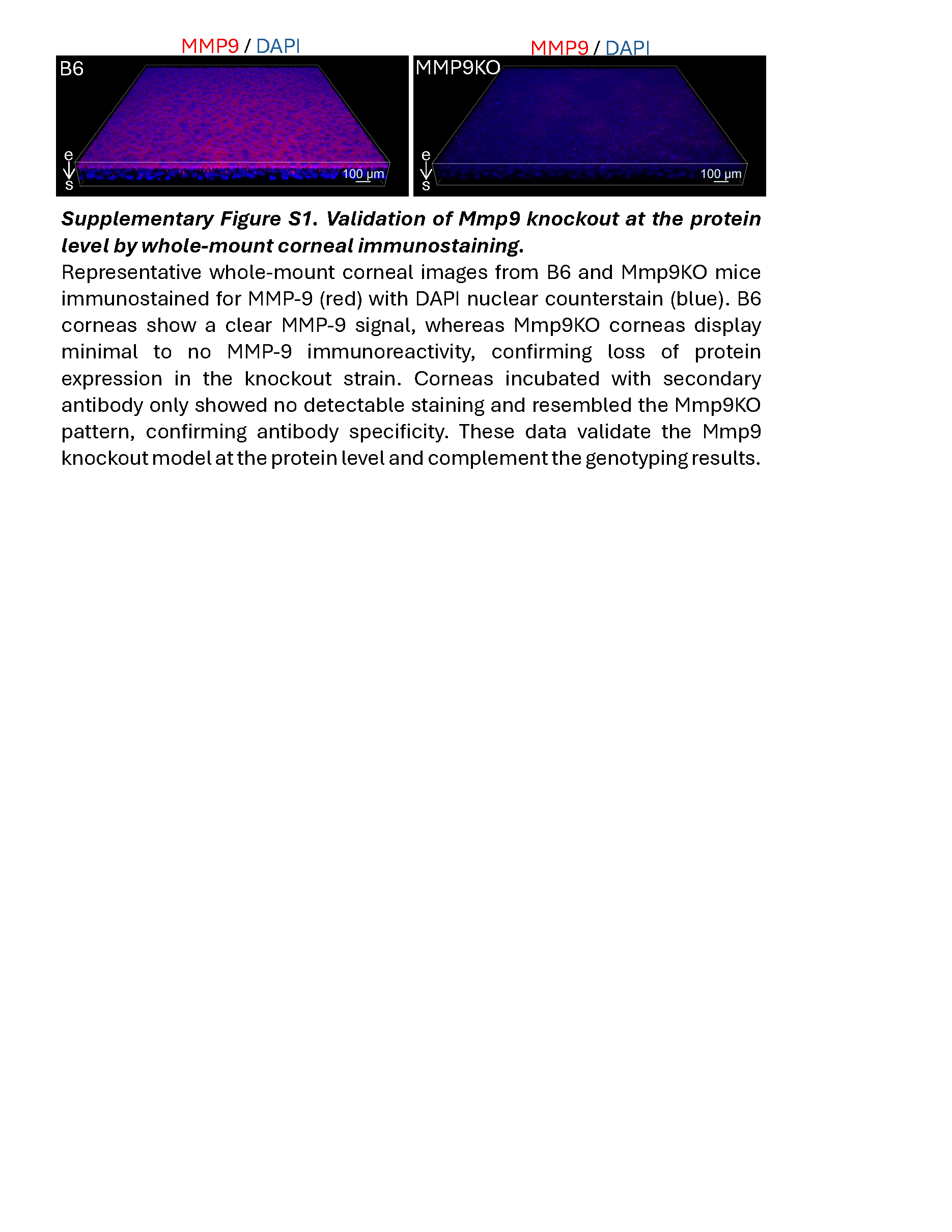

Supplement: Supplementary file 6 [file Image_1.tiff]

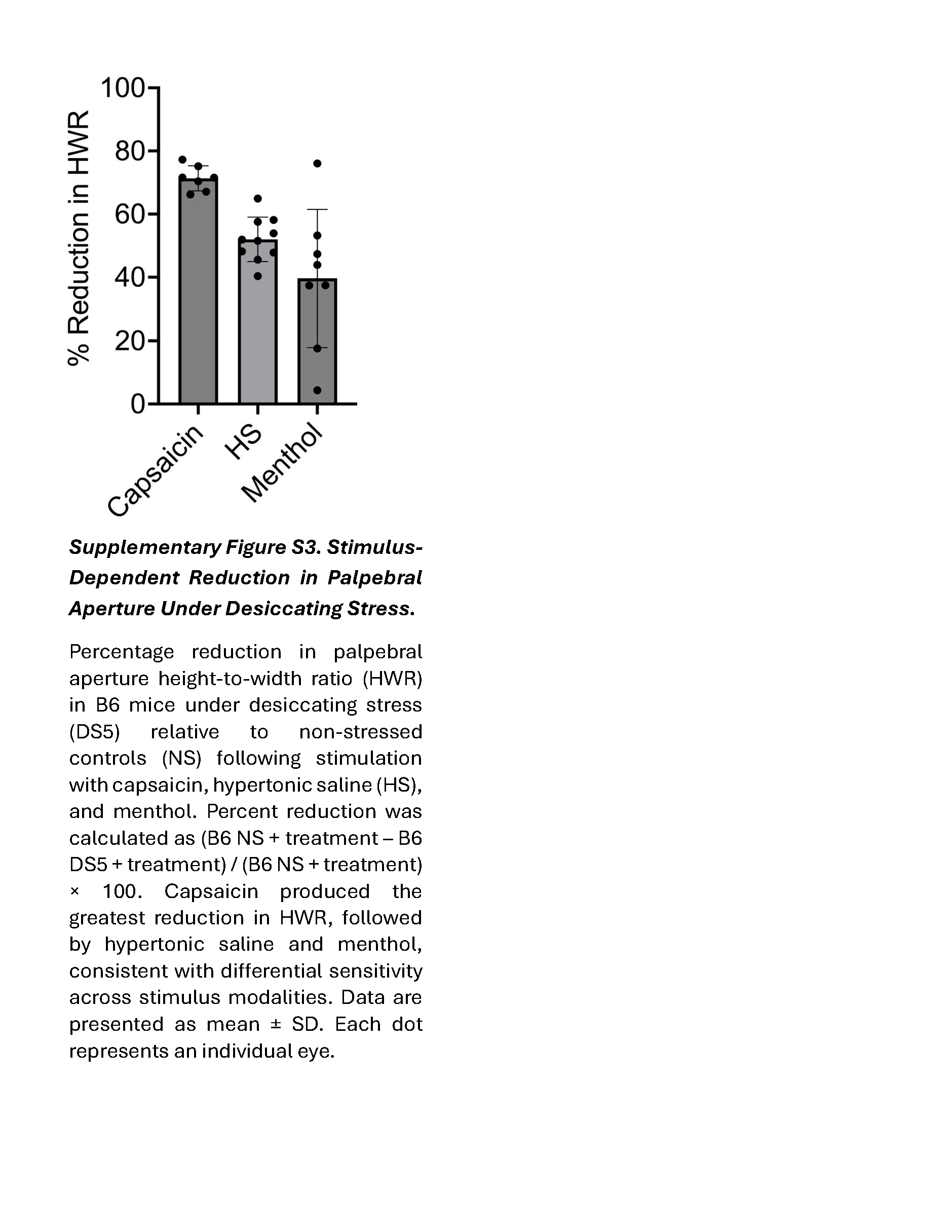

Supplement: Supplementary file 8 [file Image_3.tiff]

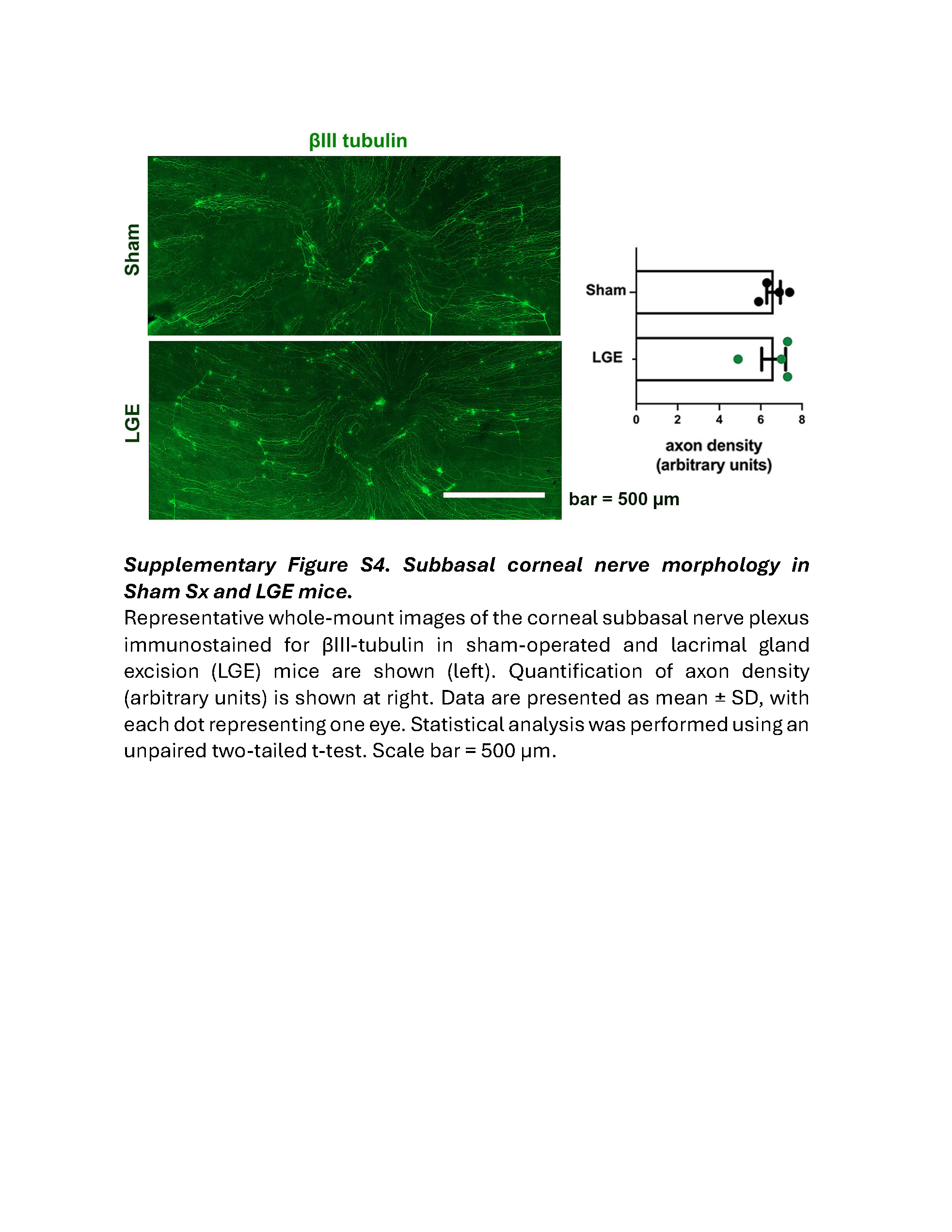

Supplement: Supplementary file 9 [file Image_4.tiff]
